# Supplementary material for: Dopamine and acetylcholine have distinct roles in delay- and effort-based decision-making in humans
Source: PLoS Biol. 2024 Jul 12;22(7):e3002714. doi: 10.1371/journal.pbio.3002714 (PMC11268711; doi:10.1371/journal.pbio.3002714)
Supplement: S1 Text — (DOCX) [file pbio.3002714.s001.docx]

# Supporting Results

## 1. Baseline Session Analysis

To analyse choice behaviour in the absence of drug manipulations, we conducted Logistic Bayesian Generalized Linear Mixed Models (GLMM) exclusively focusing on the data from the placebo (baseline) session. This analysis confirmed robust main effects of each task parameters on choice behaviour, demonstrating the expected patterns of reward and cost sensitivity across both tasks. Specifically, in both tasks, increased reward magnitudes were associated with a higher likelihood of choosing the high-cost option, while increased effort or delay levels decreased this likelihood (S1 Fig and S4 and S5 Tables).

## 2. Model Validation and Parameter Recovery

The simulated datasets mirror the original data reasonably well, confirming the model’s ability to capture essential behavioural patterns observed in the actual data (S2 Fig). Moreover, the parameter recovery revealed successful recovery of all group-level parameters, such that the mean of the simulated group-level parameters fell within the 95% HDI of the true parameter distribution (S3 Fig). Similarly, averaged simulated and actual subject-level parameters were strongly correlated (all *r* > 0.8), indicating reliable estimation at the individual subject level (S4 Fig).

## 3. Drug Effects on Vital Signs, Mood, Trail-Making Performance, MVC, and Effort Rating

Bayesian Linear Mixed Models revealed that biperiden administration led to a reduction in heart rate and systolic blood pressure at both T_1_ (heart rate: HDI_Mean_ = -6.82, HDI_95%_ = [-10.74; - 2.97]; systolic blood pressure: HDI_Mean_ = -3.91, HDI_95%_ = [-7.74; -0.13]) and T_2_ (heart rate: HDI_Mean_ = -7.88, HDI_95%_ = [-11.83; -3.89]; systolic blood pressure: HDI_Mean_ = -4.80, HDI_95%_ = [-8.74; -0.95]), with no credible impact on diastolic blood pressure (S5 Fig). Moreover, subjective ratings of alertness were credibly lower under both haloperidol and biperiden at T_2_ (biperiden: HDI_Mean_ = -0.15, HDI_95%_ = [-0.26; -0.05]; haloperidol: HDI_Mean_ = -0.15, HDI_95%_ = [-0.25; -0.04]; S6 Fig). Notably, we did not find credible drug effects on the response times in the trail-making test A, the subjective rating of effort demand, and the MVC in the effort discounting task (S7 Fig).

To investigate whether changes in physiological measures and mood ratings could be attributed to drug-induced changes in behaviour, we performed Bayesian correlation tests between each shift parameter that was credibly modulated by drug administration (i.e., *s* κ_HAL_ and *s* β_BIP_ in the delay discounting task and *s* κ_HAL_, *s* κ_BIP_, *s* β_HAL_, and *s* β_BIP_ in the effort discounting task) and the relative change in each control measure that credibly altered by drug intake (i.e., heart rate, systolic blood pressure, alertness ratings under biperiden and alertness ratings under haloperidol).

We found no credible correlation between any computational parameter that was modulated by biperiden and changes in systolic blood pressure at T_1_ in the effort (*r* = 0.10, HDI_95%_ = [-0.14; 0.33] for *s* κ_BIP_; *r* = -0.16, HDI_95%_ = [-0.39; 0.07] for *s* β_BIP_) and delay (*r* = 0.06, HDI_95%_ = [-0.19; 0.29] for *s* β_BIP_) discounting task. Similarly, we did not find any credible correlation between the computational parameters and biperiden-induced changes in systolic blood pressure at T_2_ (*r* = -0.04, HDI_95%_ = [-0.27; 0.20] for *s* κ_BIP_ (effort); *r* = -0.08, HDI_95%_ = [-0.31; 0.17] for *s* β_BIP_ (effort); *r* = 0.13, HDI_95%_ = [-0.10; 0.35] for *s* κ_BIP_ (delay)). The same applies for biperiden-induced reductions in heart rate at T_1_ (*r* = 0.05, HDI_95%_ = [-0.18; 0.28] for *s* κ_BIP_ (effort); *r* = 0.06, HDI_95%_ = [-0.18; 0.28] for *s* β_BIP_ (effort); *r* = -0.06, HDI_95%_ = [-0.29; 0.18] for *s* κ_BIP_ (delay)) and at T_2_ (*r* = 0.23, HDI_95%_ = [-0.00; 0.45] for *s* κ_BIP_ (effort); *r* = -0.09, HDI_95%_ = [-0.32; 0.16] for *s* β_BIP_ (effort); *r* = -0.18, HDI_95%_ = [-0.40; 0.07] for *s* κ_BIP_ (delay)) for both experimental paradigms. Alertness, which was affected by both drugs at T_2_, also did not show any credible correlations with biperiden- (*r* = 0.14, HDI_95%_ = [-0.10; 0.37] for *s* κ_BIP_ (effort); *r* = 0.77, HDI_95%_ = [-0.16; 0.30] for *s* β_BIP_ (effort), *r* = -0.16, HDI_95%_ = [-0.38; 0.08] for *s* β_BIP_ (delay)) as well as haloperidol-induced changes (*r* = -0.05, HDI_95%_ = [-0.28; 0.19] for *s* κ_HAL_ (effort); *r* = -0.09, HDI_95%_ = [-0.33; 0.16] for *s* β_HAL_ (effort); *r* = 0.05, HDI_95%_ = [-0.20; 0.29] for *s* κ_HAL_ (delay)).

## 4. Drug Effects on Decision Times

Having established distinct task-specific drug effects on participants’ choice behaviour, we next asked how the drugs affected the dynamics of choice, as reflected in how decision times were modulated by key decision variables. We investigated this with Bayesian Linear Mixed Models, using log-transformed decision times on each trial as the dependent variable. In both tasks, higher reward magnitudes decreased, while higher cost levels (effort or delay) increased decision times (reward effect on effort discounting: HDI_Mean_ = -0.183, HDI_95%_ = [-0.213; -0.154]; reward effect on delay discounting: HDI_Mean_ = -0.168, HDI_95%_ = [-0.190; -0.145]; effort effect on effort discounting: HDI_Mean_ = 0.084, HDI_95%_ = [0.063; 0.105]; delay effect on delay discounting: HDI_Mean_ = 0.036, HDI_95%_ = [0.020; 0.053]; S8b and S8c Fig). Notably, in both tasks, haloperidol attenuated this speeding effect of reward magnitude, indexed by a credible interaction effect between haloperidol and reward in both tasks (effort discounting: HDI_Mean_ = 0.036, HDI_95%_ = [0.009; 0.063]; delay discounting: HDI_Mean_ = 0.029, HDI_95%_ = [0.011; 0.046]: S9b and S9e Fig). Haloperidol further attenuated the decelerating effect of delay (HDI_Mean_ = -0.020, HDI_95%_ = [-0.036; -0.003]; S9f Fig), but not effort (HDI_Mean_ = -0.021, HDI_95%_ = [-0.047; 0.003]; S9c Fig). This aligns with the decreased delay sensitivity observed in the delay discounting task analysis. Moreover, haloperidol administration induced an overall increase in decision times in the delay discounting task (HDI_Mean_ = -0.077, HDI_95%_ = [-0.123; -0.031]; S9d Fig), while this effect was not observed in the effort discounting task (HDI_Mean_ = 0.016, HDI_95%_ = [-0.047; 0.078]; S9a Fig). Unlike under haloperidol, none of the effects of task parameters on response speed were modulated by biperiden. See S6 and S7 Tables for full results.

## 5. Computational Model Parameters & Self Ratings

Robust linear regression models did not reveal any significant associations between sex, self-reported questionnaire ratings (total scores and subscales), and both effort and delay baseline (placebo-condition) discounting parameters (S8 - 11 Tables). However, the analysis did reveal a significant main effect of individuals age on the effort discounting parameter, indicating a higher tendency to discount rewards in older compared to younger participants (*beta* = 0.007, *p* = 0.008).

# Supporting Materials and Methods

## 6. Fitting Procedure Bayesian Regression

To examine the impact of varying levels of reward and/or costs, as well as the administered drug, on choosing the high-reward/high-cost option, we employed Logistic Bayesian Generalized Linear Mixed Models. For the effort discounting task, the fixed effects included reward (difference between reward magnitudes of the high-cost versus low-cost option), effort (difference between effort requirement of the high-cost versus low-cost option), the administered drug (with placebo set as the reference category), and their interactions. For the delay discounting task, we followed a similar approach, but instead of using difference values, we used the absolute reward and delay levels of the high-cost option, given that the low-cost option remains fixed to a constant value throughout the task. Furthermore, to mitigate the risk of false positive results, all models contained a full random-effects structure [1]. This approach allowed us to account for individual variability, leading to more robust and reliable findings.

To control for potential confounding effects of fatigue and session, we ran additional separate GLMMs, extending the fixed-effects structures. Specifically, to test for fatigue, we included trial number and its two-way interactions with drug as additional predictors. In separate models, we controlled for session effects by adding session, as well as the two-way interactions between session and drug.

Further, to gain insights into choice behaviour in the absence of any pharmacological manipulations, we conducted additional GLMMs exclusively analysing data from the placebo sessions. These models were identical to the previously described regression analyses, including the full random-effects structure, but excluded the *drug* predictor. This analysis provided estimates of the main effects of task manipulations (i.e., reward and cost sensitivity) under the baseline condition.

To ensure robust and informative Bayesian parameter estimation and avoid issues of unstable parameter estimation that could appear with noninformative and flat priors, we followed the approach recommended by Gelman et al. and implemented weakly informative priors [2]. First, we standardized all nonbinary variables to have a mean of 0 and a standard deviation of 0.5. Then, we used the following priors in our analyses:

| Regression Coefficient | $\beta\sim Cauchy(0, 2.5)$ |
| --- | --- |
| Intercept | $\beta_{0} \sim Cauchy(0, 10)$ |
| Correlation Matrix | $\Sigma\sim LKJcorr(1)$ |
| Standard Deviation | $\sigma\sim Student(3, 0, 2.5)$ |

## 7. Fitting Procedure Hierarchical Bayesian Model

### 7.1. Model Fitting and Comparison

To identify the model that best describes how rewards are devalued by increasing levels of effort and delay, we employed four commonly used discounting models on participants’ choice data from both tasks [3–6]: linear (S1 Eq.), parabolic (S2 Eq.), hyperbolic (S3 Eq.), and exponential (S4 Eq.). To minimize the potential impact of drug administration on the discounting function, we restricted the model fitting to choice data from the (baseline) placebo condition.

$SV\left( t \right)= R(t)- \kappa*C(t)$ (1)

$SV\left( t \right)= R(t)- \kappa*{C(t)}^{2}$ (2)

$SV(t)=\frac{R(t)}{1+\exp\left( \kappa\right)*C(t)}$ (3)

$SV\left( t \right)= R\left( t \right)*e^{-\kappa*C(t)}$ (4)

All discounting models assume that the subjective value (SV) of an offer is calculated by taking into account the reward (R) and the cost (C) level on trial (t). In the effort discounting task, the cost level is represented by the effort level, which is scaled to the proportion of the maximum voluntary contraction. In the delay discounting task, the cost level is represented by the delay of the high-cost option, indicating the number of days necessary to wait to obtain the reward. The degree to which rewards are discounted by increasing levels of costs is modelled by a subject-specific discounting parameter (κ), which quantifies the steepness of each individual's devaluation of rewards as costs increase. Higher values of κ represent higher steepness in devaluation, indicating stronger sensitivity to increasing costs, while lower values represent lower steepness, indicating less sensitivity to increasing costs. Importantly, in the effort discounting task, two options with varying levels of reward and effort are presented on each trial, leading to the calculation of two different SVs. In contrast, in the delay discounting task, one option varies in reward and delay, while the other SV is fixed at 20, resulting in the calculation of a single SV for that option per trial. Additionally, note that the κ values for the delay discounting task were modelled in log space to prevent numerical instability caused by highly skewed κ values. SVs for the high-reward/high-cost (HC) and the low-reward/low-cost (LC) were then transformed to choice probabilities, using a softmax function (S5 Eq.).

$P\left( {HC}_{(t)} \right)= \frac{exp(SV({HC}_{(t)})* \beta)}{\exp\left( SV({HC}_{(t)})* \beta\right)+exp(SV({LC}_{(t)})* \beta)}$ (5)

The choice consistency was modelled using the inverse temperature parameter β.

To determine the best fitting models for describing participants' behaviour in each task, we employed the leave-one-out information criterion (LOOIC), which estimates out-of-sample prediction accuracy by utilizing the log-likelihood. The LOOIC was assessed using the loo package in R [7]. Lower LOOIC values indicate better model fit, similar to traditional information criteria such as AIC and BIC (see S3 Table).

### 7.2. Model Parametrization and Priors

For all hierarchical models, we assume that the subject-level parameters are drawn from group-level normal distributions. We use Uniform and half-Cauchy distributions for the group-level mean (μ) and standard deviations (σ) of the (baseline) placebo-condition discounting parameters κ and inverse temperature β, respectively. For all shift parameters, which indicate drug-specific effects on κ and β, Gaussian prior distributions were used for the means, and half-Cauchy distributions were used for the standard deviations. Based on previous findings, the standard deviations of all half-Cauchy distributions were set with a location of 0 and a scale of 2.5. Additionally, more restrictive priors were set for the Gaussian distribution of all group-level shift hyperparameters with a location of 0 and a scale of 2 [8–10]. To account for the different degrees of discounting depending on the cost type and the logarithmic transformation of κ in delay discounting, we used distinct ranges for the Uniform distribution of the group-level means in both tasks. These ranges were based on numerically plausible values and previous findings [8–12].

In summary, the prior distributions for our hierarchical models are as follows:

$$\mu_{\kappa(Effort)} \sim Uniform(0, 5)$$

$$\mu_{\kappa(Delay)} \sim Uniform(-20, 3)$$

$$\sigma_{\kappa} \sim HalfCauchy(0, 2.5)$$

$$\mu_{\beta} \sim Uniform(0, 10)$$

$$\sigma_{\beta} \sim HalfCauchy(0, 2.5)$$

$$\mu_{S_{x}} \sim Normal(0, 2)$$

$$\sigma_{S_{x}} \sim HalfCauchy(0, 2.5)$$

## 8. Physiological Measures and Bond-Lader Visual Analogue Scale

Participants completed subjective mood ratings using the German version of the Bond and Lader visual analogue scale [13] at three different time points (T_0_, T_1_, and T_2_). Additionally, blood pressure and heart rate measurements were taken at the same time points using a digital blood pressure monitor (OMRON model M500, Healthcare Europe B.V., The Netherlands). These manipulation checks were conducted at T_0_ (before drug administration), T_1_ (before starting the task, approximately 170 min after haloperidol or 50 min after biperiden intake), and T_2_ (after finishing the task, approximately 230 min after haloperidol or 110 min after biperiden intake). These assessments allowed us to examine potential effects of the administered drugs on subjective mood states and physiological parameters. Subjective mood rating scales involved 16 binary items presented on a horizontal line on a sheet of paper. Each item consisted of two words describing opposing mood states (e.g., “happy versus sad”), and participants indicated their mood by marking the line closer to one of the two words. Based on a factor analysis using a principal component solution and orthogonal rotation of the factor matrix, three separate factor scores were extracted: alertness, contentedness, and calmness. Self-ratings were analysed by measuring the distance in millimetres from the end of the line to the subject's mark. These measurements were then log-transformed to correct for skewness [13]. In addition, once per session, participants completed an effort rating, in which they were required to rate each effort level they encountered during the tasks. Furthermore, prior to beginning the effort discounting task, participants completed the trail-making test A, and we measured participants’ maximum voluntary contraction (MVC).

To analyse the effects of the drugs on mood ratings, physiological measures, trail-making test response times, MVC, and subjective effort perception ratings, we applied Bayesian Linear Mixed Effects Models. For mood ratings and physiological measures, the models included the factors time (T_0_, T_1_, and T_2_), drug (PLC, HAL, BIP), and their interaction as fixed effects, with subject-specific intercepts. Similarly, for the trail-making test, MVC, and effort ratings, which were measured once per session, the models included drug (PLC, HAL, BIP), session (Session 1, Session 2, Session 3), and their interaction as fixed effects, with subject-specific intercepts. For analysing effort ratings, we additionally included effort levels as fixed effects in the model. For parameter estimation, we used non-informative priors (the brms default) and ran four chains with 3000 samples (1000 samples for warmup).

Importantly, to test whether credible changes in physiological parameters or mood ratings induced by drug administration could explain changes in behaviour, we used Bayesian correlation tests to examine possible associations. We correlated difference values (T_0_ vs. timepoints with credible drug-induced influences; i.e., T_2_ for mood ratings, T_1_ and T_2_ for physiological parameters) with the mean estimates of all shift parameters that were credibly modulated by either haloperidol or biperiden.

## 9. Decision Times Analysis

Similarly, to the model-agnostic analysis of the choice data, we performed a separate analysis focusing on participants' decision times in both tasks using Bayesian Linear Mixed Models. In contrast to the previous analysis, with binary choice data as the outcome variable with a Bernoulli response distribution and a logit link function, here, we used the log-transformed decision times (in milliseconds) as the outcome variable with a Gaussian distribution function. We then regressed the decision times to the same set of fixed-effect predictors, including drug, reward, cost (i.e., delay or effort), and their respective interaction terms.

However, in order to ensure full model convergence, we reduced the random-effects structure (the full random-effects structure led to convergence issues, indicated by r-hat values > 1.05). Specifically, we removed the three-way interaction effect (interaction between drug, reward, and cost type), including only main and two-way interaction effects in the random effects structure. As mentioned earlier, we applied weakly informative priors, scaling nonbinary variables to have a mean of 0 and a standard deviation of 0.5. Posterior distributions of parameter estimates were obtained by running four chains with 3000 samples, including 1000 samples for warmup.

## 10. Computational Parameter Estimates, Self-Rating Scores, and Demographics

As a last step of our analysis, we examined potential associations between the discounting parameters κ and self-reported questionnaire ratings, including the Apathy Evaluation Scale (AES) and Barratt Impulsiveness Scale-15 (BIS-15), with all subscales, and Beck Depression Inventory (BDI). We also investigated the relationship of both model parameters with demographic variables (sex and age). To this aim, we conducted four separate robust linear regressions, using the mean estimates of the discounting parameter κ from the placebo condition of both tasks, serving as a baseline value for each participants’ discounting behaviour. We chose robust regression models because they have been shown to be less sensitive to the influence of outliers [14]. We regressed this outcome against predictors of age, sex, and the z-scored total scores of all questionnaire scores. Further, to gain insights into the effects of each subscale of the apathy and impulsivity questionnaires, we again performed separate robust regression models, this time using the z-scored subscale scores of both questionnaires as predictors.

# Supporting References

1. Barr DJ, Levy R, Scheepers C, Tily HJ. Random effects structure for confirmatory hypothesis testing: Keep it maximal. J Mem Lang. 2013;68. doi:10.1016/j.jml.2012.11.001

2. Gelman A, Jakulin A, Pittau MG, Su Y-S. A weakly informative default prior distribution for logistic and other regression models. aoas. 2008;2: 1360–1383. doi:10.1214/08-AOAS191

3. Białaszek W, Marcowski P, Ostaszewski P. Physical and cognitive effort discounting across different reward magnitudes: Tests of discounting models. PLoS One. 2017;12: e0182353. doi:10.1371/journal.pone.0182353

4. Chong TT-J, Apps M, Giehl K, Sillence A, Grima LL, Husain M. Neurocomputational mechanisms underlying subjective valuation of effort costs. PLoS Biol. 2017;15: e1002598. doi:10.1371/journal.pbio.1002598

5. Hartmann MN, Hager OM, Tobler PN, Kaiser S. Parabolic discounting of monetary rewards by physical effort. Behav Processes. 2013;100: 192–196. doi:10.1016/j.beproc.2013.09.014

6. Klein-Flügge MC, Kennerley SW, Saraiva AC, Penny WD, Bestmann S. Behavioral modeling of human choices reveals dissociable effects of physical effort and temporal delay on reward devaluation. PLoS Comput Biol. 2015;11: e1004116. doi:10.1371/journal.pcbi.1004116

7. Vehtari A, Gelman A, Gabry J. Practical Bayesian model evaluation using leave-one-out cross-validation and WAIC. Stat Comput. 2017;27: 1413–1432. doi:10.1007/s11222-016-9696-4

8. Knauth K, Peters J. Trial-wise exposure to visual emotional cues increases physiological arousal but not temporal discounting. Psychophysiology. 2022;59: e13996. doi:10.1111/psyp.13996

9. Mathar D, Erfanian Abdoust M, Marrenbach T, Tuzsus D, Peters J. The catecholamine precursor Tyrosine reduces autonomic arousal and decreases decision thresholds in reinforcement learning and temporal discounting. PLoS Comput Biol. 2022;18: e1010785. doi:10.1371/journal.pcbi.1010785

10. Wagner B, Clos M, Sommer T, Peters J. Dopaminergic Modulation of Human Intertemporal Choice: A Diffusion Model Analysis Using the D2-Receptor Antagonist Haloperidol. J Neurosci. 2020;40: 7936–7948. doi:10.1523/JNEUROSCI.0592-20.2020

11. Lockwood PL, Abdurahman A, Gabay AS, Drew D, Tamm M, Husain M, et al. Aging Increases Prosocial Motivation for Effort. Psychol Sci. 2021; 956797620975781. doi:10.1177/0956797620975781

12. Lockwood PL, Wittmann MK, Nili H, Matsumoto-Ryan M, Abdurahman A, Cutler J, et al. Distinct neural representations for prosocial and self-benefiting effort. Curr Biol. 2022. doi:10.1016/j.cub.2022.08.010

13. Bond A, Lader M. The use of analogue scales in rating subjective feelings. Br J Med Psychol. 1974;47: 211–218. doi:10.1111/j.2044-8341.1974.tb02285.x

14. Yu C, Yao W. Robust linear regression: A review and comparison. Communications in Statistics - Simulation and Computation. 2017;46: 6261–6282. doi:10.1080/03610918.2016.1202271
