# Supplementary figures and images for: Dopamine and acetylcholine have distinct roles in delay- and effort-based decision-making in humans
Source: PLoS Biol. 2024 Jul 12;22(7):e3002714. doi: 10.1371/journal.pbio.3002714 (PMC11268711; doi:10.1371/journal.pbio.3002714)

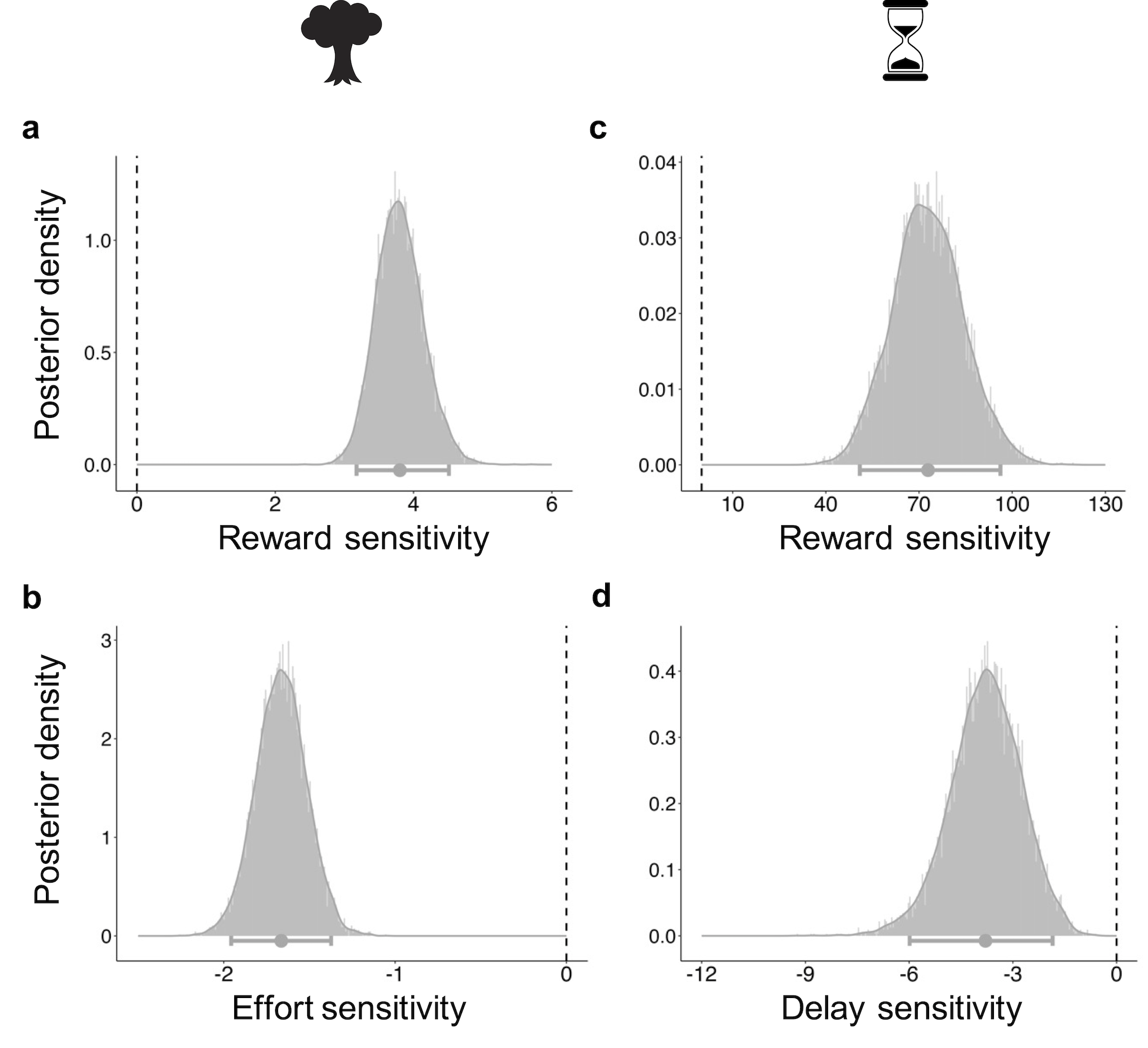

Supplement: S1 Fig — Posterior distributions and 95% HDI of the logistic Bayesian generalized linear mixed models depict the estimate of each task parameter on choosing the high-cost option. (a) Higher reward magnitudes increased the overall willingness to invest physical effort for a corresponding reward in the effort discounting task. (b) Higher levels of effort had the opposite effect. (c) Similarly, higher reward magnitudes increased the likelihood to choose the high-cost option in the delay discounting task. (d) In contrast, higher levels of delay decreased the willingness to choose the high-reward/high-delay option. Bold dots represent the mean group-level estimate of the posterior distribution. The horizontal bars represent the group-level 95% highest density interval. (TIF) [file pbio.3002714.s004.tif]

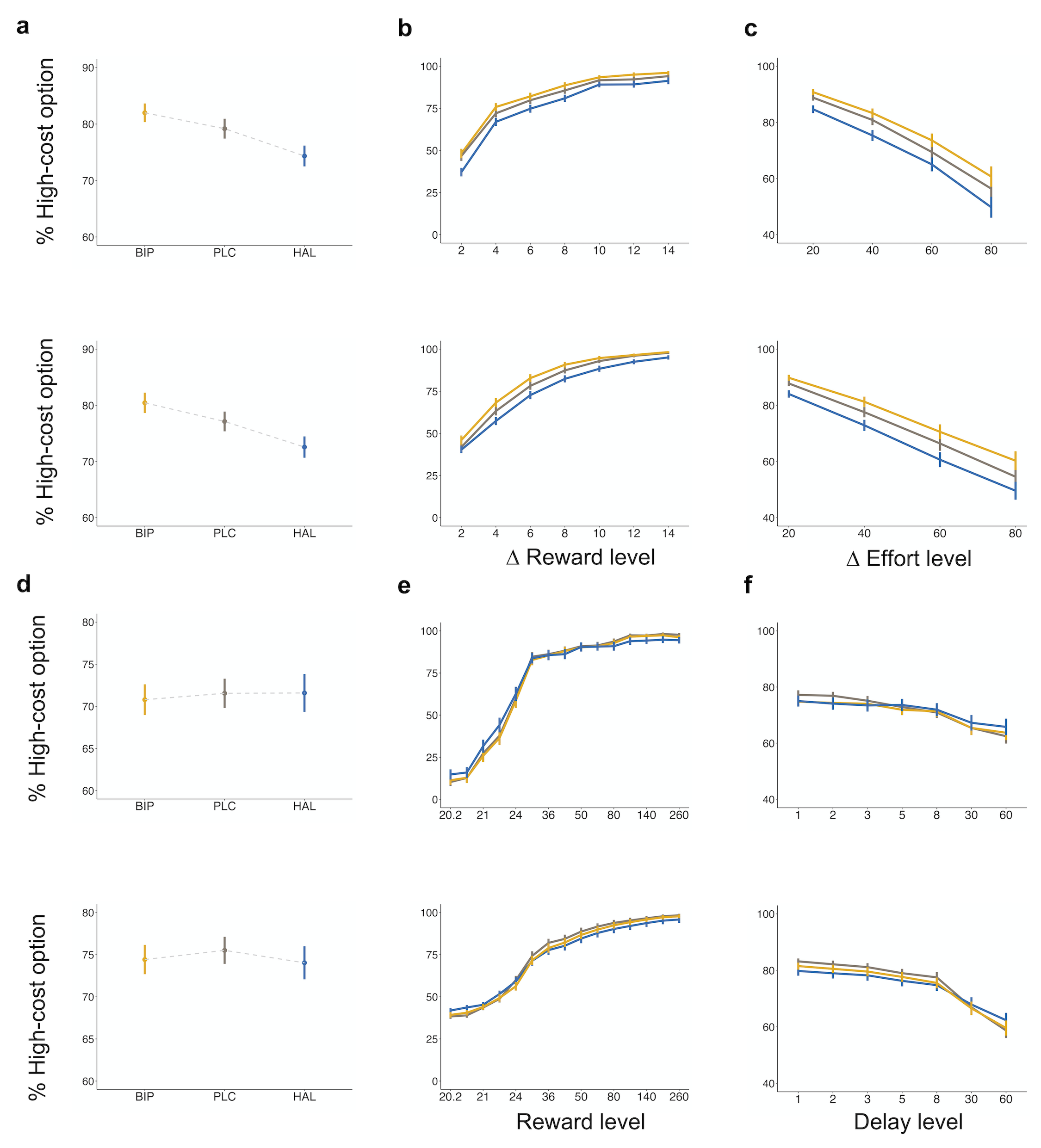

Supplement: S2 Fig — Model validation for the effort (a–c) and delay discounting task (d–f). Plots depict the averaged overall proportion of choosing the high-cost option as a function of reward and cost for both the effort (a–c) and the delay (d–f) discounting task. The upper panels display the actual data and the lower panels present the simulated data for comparison. Group-level means are indicated by dots, with error bars representing the standard error of the mean. In the effort discounting task, reward levels are presented as the difference in magnitude between the high- and low-cost option, and in the delay discounting task, reward levels are shown as the reward value of the high-cost option. Likewise, the effort level corresponds to the difference between the proportions of the individually calibrated MVC of the high- and low-cost option, while the delay levels indicate the delay of the high-cost option. (TIF) [file pbio.3002714.s005.tif]

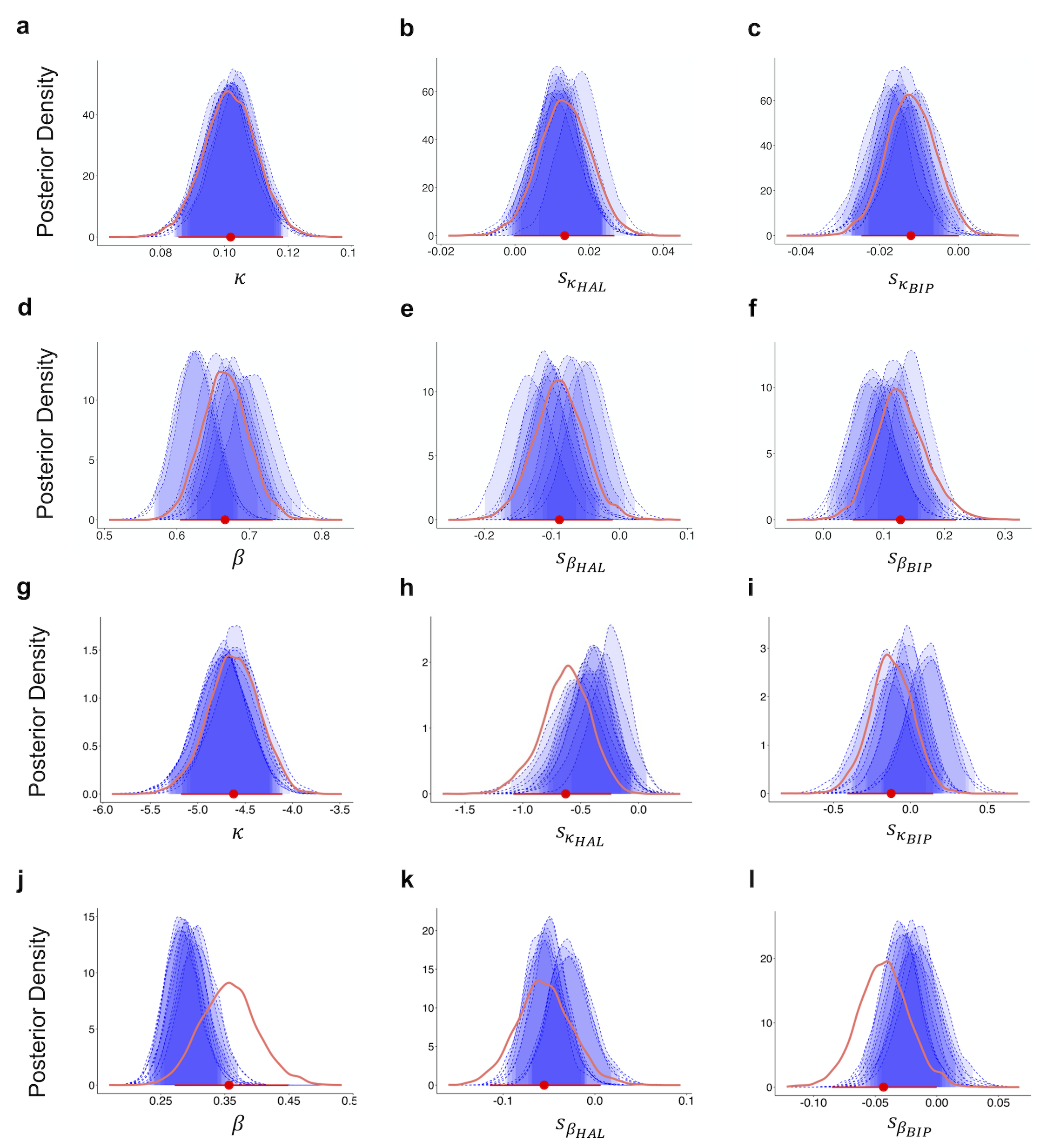

Supplement: S3 Fig — Parameter recovery of the group-level posterior distributions for the effort (a–f) and delay discounting task (g–l). We tested the ability of our models to recover parameters using simulated datasets. Each panel displays the actual distribution (red) of each parameter of interest alongside 10 corresponding simulated datasets (blue). Horizontal bars, depicted in red, represent the group-level 95% HDI of the actual parameter estimate, with dots indicating the mean of the distribution. The shaded area in blue depicts the 95% HDI of the simulated parameter estimates. We evaluated whether the mean estimates of the simulated group-level parameter values fell within the 95% HDI of the true parameter distribution. The parameter recovery analysis of the group-level distribution demonstrated positive results, as all mean parameter estimates of the simulated data are located within the 95% HDI of the actual dataset. (TIF) [file pbio.3002714.s006.tif]

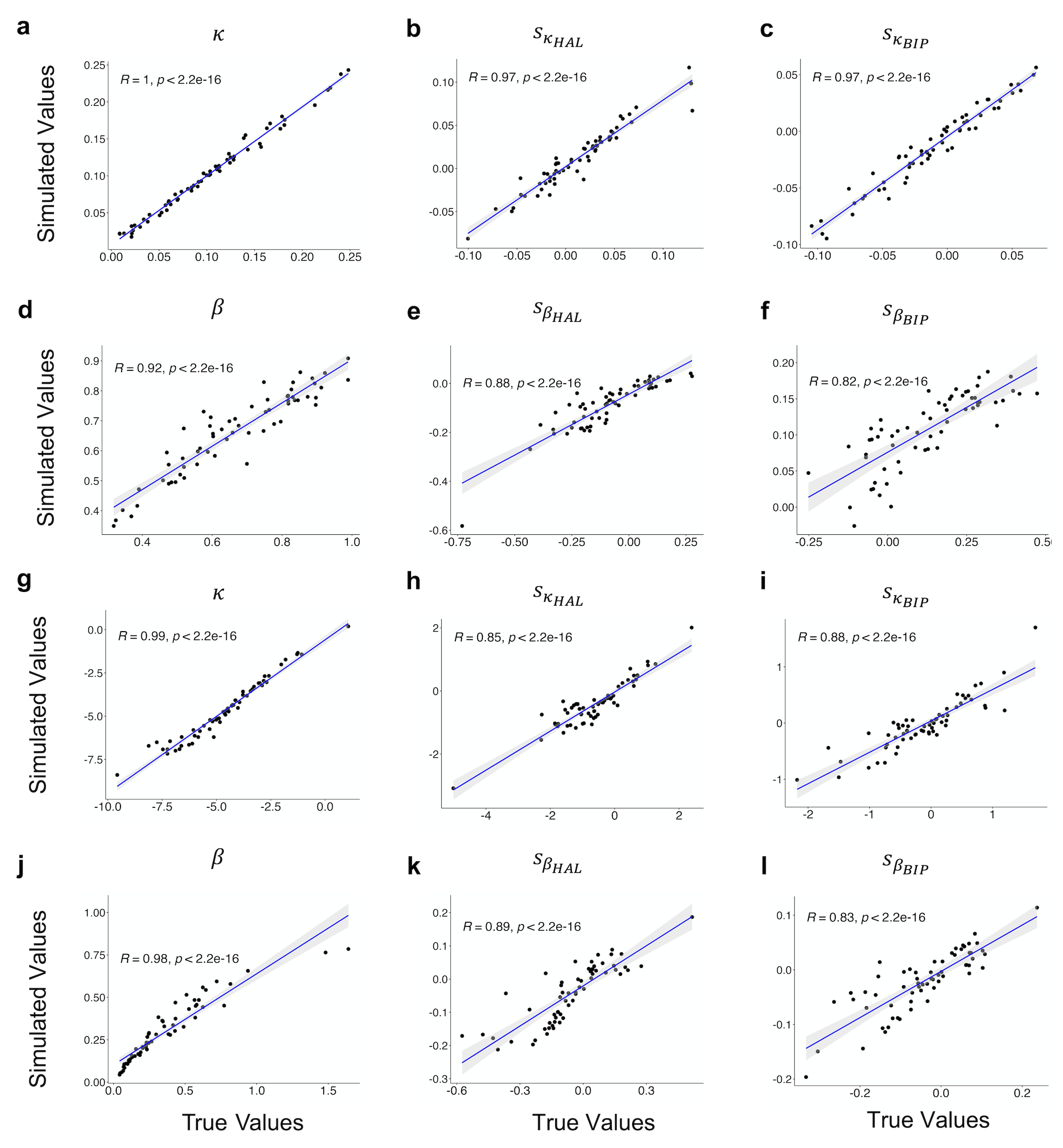

Supplement: S4 Fig — Parameter recovery of the subject-level parameters for the effort (a–f) and delay discounting task (g–l). We calculated the Pearson correlation coefficients between the mean subject-level posterior distribution of the simulated and actual data. For the simulated data, subject-level means were averaged across each dataset. The correlation coefficients demonstrate strong to excellent correlations (all r > 0.8), further confirming that the models are able to accurately to recover the actual task parameter values. (TIF) [file pbio.3002714.s007.tif]

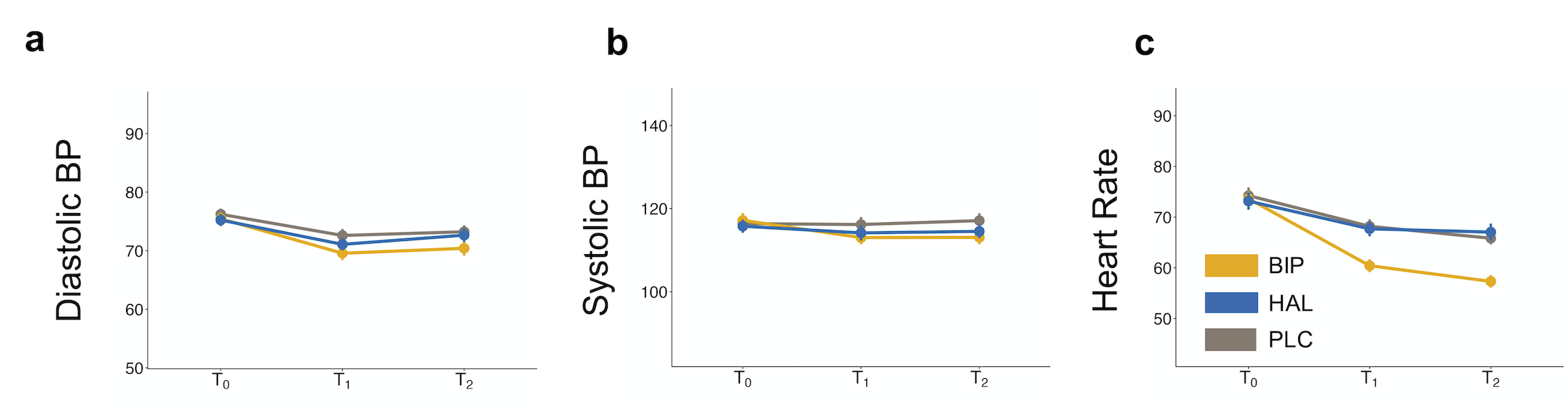

Supplement: S5 Fig — Plots depict the change in each physiological parameter following drug administration. (a) Diastolic BP decreased at T1 (HDIMean = −3.75, HDI95% = [−5.71; −1.85]) and T2 (HDIMean = −2.99, HDI95% = [−4.96; −1.07]) compared to T0. However, we did not find a credible main or interaction effect of drug, implying that diastolic BP decreases as the task progresses irrespective of drug administration. (b) Systolic BP reflected notable two-way biperiden interactions at T1 and T2 (Biperiden x T1: HDIMean = −3.91, HDI95% = [−7.74; −1.07]; Biperiden x T2: HDIMean = −4.80, HDI95% = [−8.74; −0.95]), suggesting a more pronounced decrease in systolic BP following Biperiden application. (c) Heart rate was reduced at T1 (HDIMean = −6.82, HDI95% = [−10.74; −2.97]) and T2 (HDIMean = −7.88, HDI95% = [−11.83; −3.89]) relative to T0. A credible two-way interaction was found between Biperiden at T1 and T2 (Biperiden x T1: HDIMean = −6.82, HDI95% = [−10.74; −2.97]; Biperiden x T2: HDIMean = −7.88, HDI95% = [−11.83; −3.89]), indicating that, analogously to the drop in systolic BP, the drop in heart rate at T1 and T2 is more pronounced following biperiden administration compared to placebo. Dots represent the group-level mean, error bars depict the standard error of the mean. (TIF) [file pbio.3002714.s008.tif]

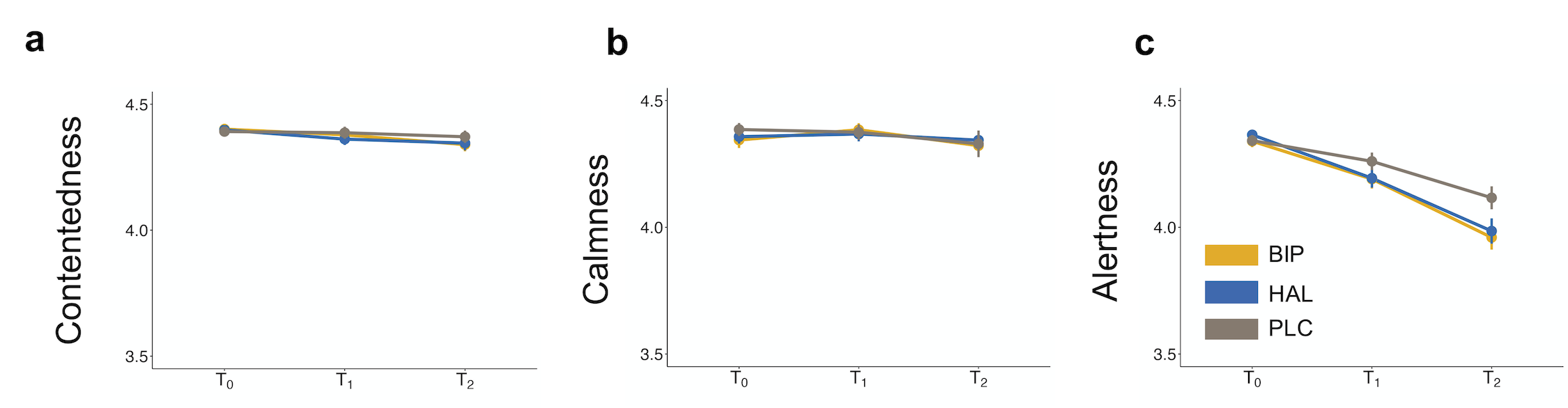

Supplement: S6 Fig — Plots show alterations in subjective mood ratings following drug administration. No credible effects of time or drug were found for (a) contentedness and (b) calmness ratings. However, (c) alertness ratings notably decreased over time, exhibiting a credible drop at both T1 (HDIMean = −0.08, HDI95% = [−0.16; −0.01]) and T2 (HDIMean = −0.23, HDI95% = [−0.30; −0.15]) compared to T0. Importantly, at T2, credible two-way interaction effects were found for biperiden and haloperidol (Biperiden x T2: HDIMean = −0.15, HDI95% = [−0.26; −0.05]; Haloperidol x T2: HDIMean = −0.15, HDI95% = [−0.25; −0.04]), suggesting that both drugs led to more pronounced reductions in alertness ratings towards the end of the experiment, compared to placebo. Dots represent the group-level mean, error bars depict the standard error of the mean. (TIF) [file pbio.3002714.s009.tif]

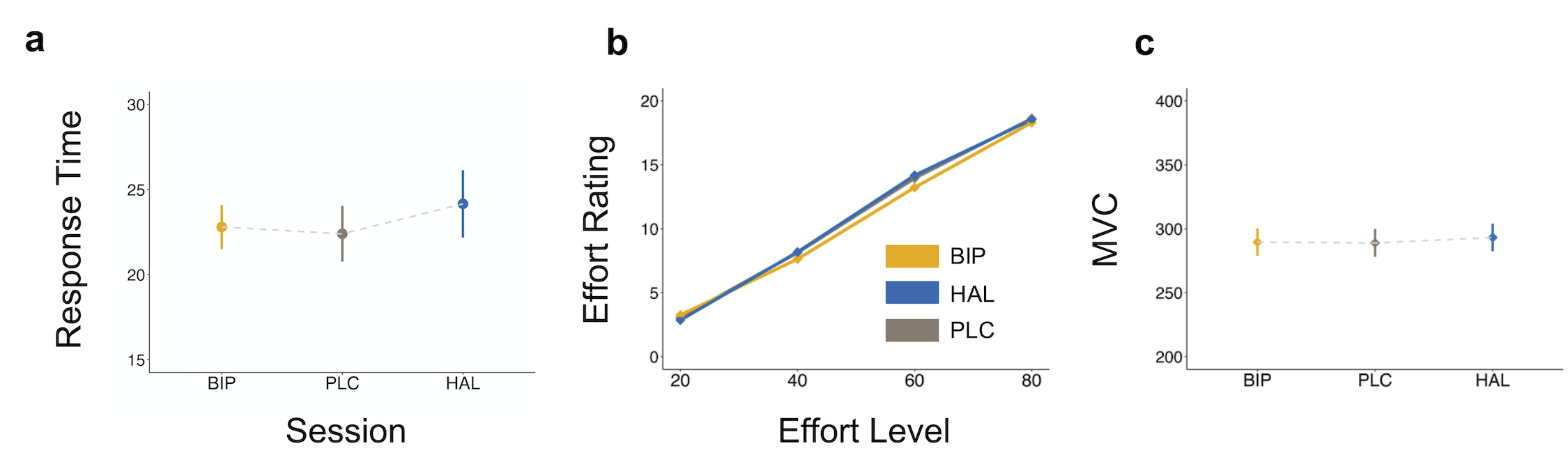

Supplement: S7 Fig — (a) No credible drug effects were observed for response times during trail-making test A. However, credible main effects of session were found (Session 2: HDIMean = −4.77, HDI95% = [−8.41; −1.17]; Session 3: HDIMean = −6.40, HDI95% = [−10.14; −2.66]), suggesting that participants became faster after the first session, possibly due to familiarity with the task. (b) Effort ratings were modulated only by increasing effort levels (HDIMean = 5.15, HDI95% = [4.81; 5.49]), suggesting no drug effects on the subjective experience of effort demand. (c) MVC was not credibly modulated by drug administration, indicating that participant’s ability to exert effort was not modulated by drug either. Dots represent the group-level mean and error bars represent standard error of the mean. (TIF) [file pbio.3002714.s010.tif]

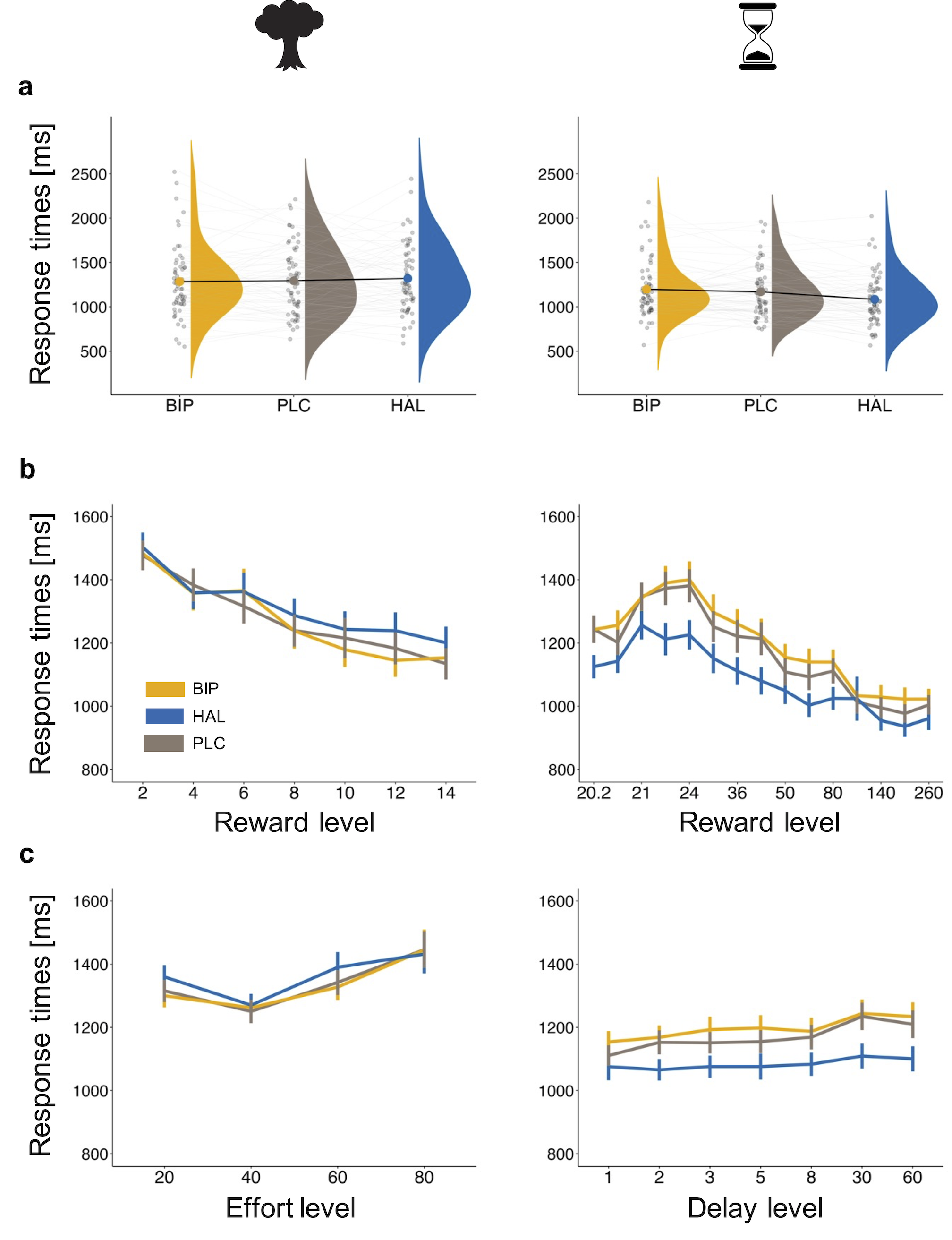

Supplement: S8 Fig — Decision times in milliseconds (ms) in the effort (left panel) and delay (right panel) discounting tasks. (a) Overall decision times in the effort discounting task (left) were not affected by haloperidol or biperiden. However, in the delay discounting task (right), haloperidol reduced the decision times. (b) Decision times decreased with larger rewards. Haloperidol reduced this speed-up effect in both the effort (left) and delay discounting task (right panel). (c) Conversely, decision times increased with higher cost levels. This effect was not modulated by any drug in the effort discounting task (left). However, in the delay discounting task (right), haloperidol diminished the decelerating effect of increasing delay levels. (a) Shows group-level (single-subject) means represented by bold (light) dots. (b and c) Display averaged group-level means per reward and cost level, with error bars representing the standard error of the mean. Reward levels are presented as the difference in magnitude between the high- and low-cost option in the effort discontinuing task and as the absolute reward value of the high-cost option in the delay discounting task. Likewise, the effort level represents the difference between the proportions of the individually calibrated MVC of the high- and low-cost option, while the delay level indicates the delay of the high-cost option. The data underlying the effort discounting task (left panel) can be found in S1 Data, and the data underlying the delay discounting task (right panel) can be found in S2 Data. (TIF) [file pbio.3002714.s011.tif]

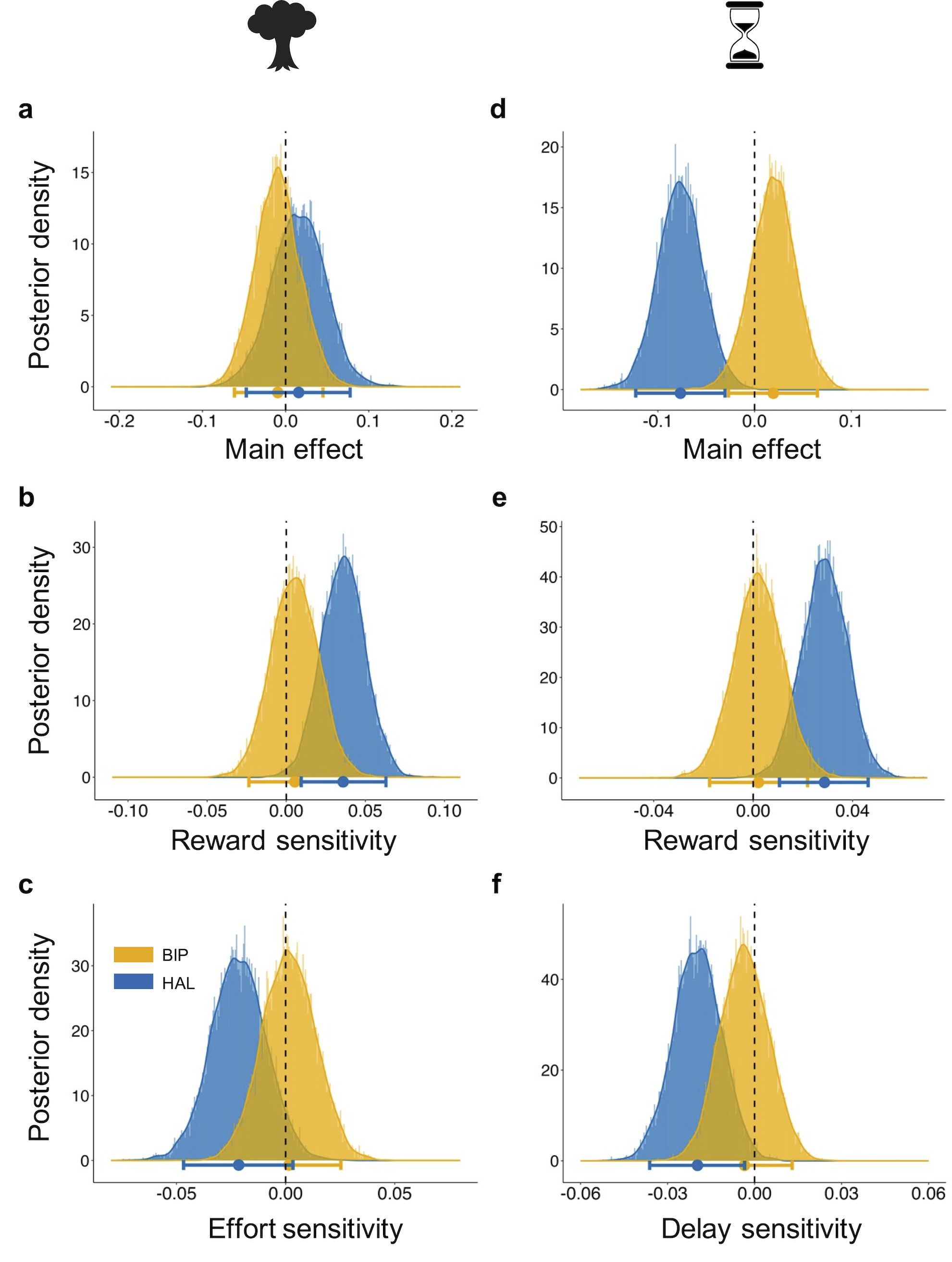

Supplement: S9 Fig — Posterior distributions and 95% HDI of the Bayesian linear mixed models depict the estimate of each effect on decision times. (a) In the effort discounting task, overall decision times were not credibly influenced by either haloperidol or biperiden. (b) Notably, a haloperidol-by-reward interaction revealed a reduced reward sensitivity after haloperidol administration. (c) On the other hand, sensitivity towards increasing levels of effort is not affected by either drug. (d) In the delay discounting task, haloperidol credibly reduced overall decision times, while biperiden did not show any credible effect. (e) Analogous to the effort discounting task, a credible haloperidol-by-reward interaction demonstrate reduced reward sensitivity following haloperidol administration. (f) Furthermore, haloperidol administration credibly reduced delay sensitivity, while biperiden had no effect on the impact of delay on decision times. Bold dots represent the mean group-level estimate of the posterior distribution. The horizontal bars represent the group-level 95% HDI. (TIF) [file pbio.3002714.s012.tif]
