# Supplementary material for: Dopamine and acetylcholine have distinct roles in delay- and effort-based decision-making in humans
Source: PLoS Biol. 2024 Jul 12;22(7):e3002714. doi: 10.1371/journal.pbio.3002714 (PMC11268711; doi:10.1371/journal.pbio.3002714)
Supplement: S1 Table — (DOCX) [file pbio.3002714.s013.docx]

**S1 Table.** Bayesian Generalized Linear Mixed Models of the Effort Discounting Task, Regressing Choices (High-Cost vs. Low-Cost Option) on Predictors for Drug, Reward (Difference between High-Cost vs. Low-Cost Reward Level), Effort (Difference between High-Cost vs. Low-Cost Effort Level), and their Interaction Terms.

| **Parameter** | **Estimate** | **Est. Error** | **2.5%** | **97.5%** |
| --- | --- | --- | --- | --- |
| **(Intercept)** | 2.544 | 0.219 | 2.116 | 2.988 |
| **Biperiden** | 0.620 | 0.207 | 0.230 | 1.048 |
| **Haloperidol** | -0.532 | 0.203 | -0.943 | -0.136 |
| **Reward** | 3.436 | 0.251 | 2.961 | 3.945 |
| **Effort** | -1.638 | 0.122 | -1.874 | -1.402 |
| **Biperiden x Reward** | 0.802 | 0.292 | 0.256 | 1.409 |
| **Haloperidol x Reward** | -0.296 | 0.257 | -0.797 | 0.203 |
| **Biperiden x Effort** | -0.011 | 0.153 | -0.309 | 0.303 |
| **Haloperidol x Effort** | 0.084 | 0.121 | -0.150 | 0.321 |
| **Reward x Effort** | 0.150 | 0.183 | -0.212 | 0.509 |
| **Biperiden x Reward x Effort** | 0.104 | 0.295 | -0.476 | 0.675 |
| **Haloperidol x Reward x Effort** | -0.223 | 0.238 | -0.682 | 0.256 |
