# Supplementary material for: Dopamine and acetylcholine have distinct roles in delay- and effort-based decision-making in humans
Source: PLoS Biol. 2024 Jul 12;22(7):e3002714. doi: 10.1371/journal.pbio.3002714 (PMC11268711; doi:10.1371/journal.pbio.3002714)
Supplement: S2 Table — (DOCX) [file pbio.3002714.s014.docx]

**S2 Table.** Bayesian Generalized Linear Mixed Models of the Delay Discounting Task, Regressing Choices (High-Cost vs. Low-Cost Option) on Predictors for Drug, Reward (High-Cost Option Reward), Delay (High-Cost Option Delay), and their Interaction Terms.

| **Parameter** | **Estimate** | **Est. Error** | **2.5%** | **97.5%** |
| --- | --- | --- | --- | --- |
| **(Intercept)** | 19.555 | 2.629 | 14.436 | 24.643 |
| **Biperiden** | -1.315 | 1.263 | -4.147 | 0.655 |
| **Haloperidol** | -0.046 | 1.095 | -2.648 | 1.884 |
| **Reward** | 55.637 | 7.095 | 41.762 | 69.382 |
| **Delay** | -2.291 | 0.522 | -3.303 | -1.250 |
| **Biperiden x Reward** | -3.871 | 3.478 | -11.873 | 1.453 |
| **Haloperidol x Reward** | -1.271 | 2.952 | -8.257 | 4.037 |
| **Biperiden x Delay** | 0.781 | 0.481 | -0.104 | 1.807 |
| **Haloperidol x Delay** | 1.332 | 0.540 | 0.328 | 2.440 |
| **Reward x Delay** | -2.804 | 1.382 | -5.521 | -0.142 |
| **Biperiden x Reward x Delay** | 1.054 | 1.333 | -1.361 | 4.003 |
| **Haloperidol x Reward x Delay** | 2.383 | 1.525 | -0.465 | 5.514 |
