# Supplementary material for: Dopamine and acetylcholine have distinct roles in delay- and effort-based decision-making in humans
Source: PLoS Biol. 2024 Jul 12;22(7):e3002714. doi: 10.1371/journal.pbio.3002714 (PMC11268711; doi:10.1371/journal.pbio.3002714)
Supplement: S3 Table — (DOCX) [file pbio.3002714.s015.docx]

**S3 Table.** Model comparison for the effort and delay discounting task. To compare the validity of each model, we used the leave-one-out cross-validation information criterion (LOOIC) procedure. A lower LOOIC score indicates a better-fitting model.

| **Model** | ***Effort*** | ***Delay*** |
| --- | --- | --- |
|  | **LOOIC** | **LOOIC** |
| *Parabolic* | 15109.7* | 27279.6 |
| *Linear* | 17337.5 | 26041.5 |
| *Hyperbolic* | 18477.9 | 25662.4* |
| *Exponential* | 17961.2 | 26217.5 |
