# Supplementary material for: Dopamine and acetylcholine have distinct roles in delay- and effort-based decision-making in humans
Source: PLoS Biol. 2024 Jul 12;22(7):e3002714. doi: 10.1371/journal.pbio.3002714 (PMC11268711; doi:10.1371/journal.pbio.3002714)
Supplement: S4 Table — (DOCX) [file pbio.3002714.s016.docx]

**S4 Table.** Bayesian Generalized Linear Mixed Models of the Effort Discounting Task – Baseline Session; Regressing Choices (High-Cost vs. Low-Cost Option) on Predictors for Reward (Difference between High-Cost vs. Low-Cost Reward Level), Effort (Difference between High-Cost vs. Low-Cost Effort Level), and their Interaction Terms.

| **Parameter** | **Estimate** | **Est. Error** | **2.5%** | **97.5%** |
| --- | --- | --- | --- | --- |
| **(Intercept)** | 2.747 | 0.288 | 2.199 | 3.331 |
| **Reward** | 3.802 | 0.342 | 3.174 | 4.512 |
| **Effort** | -1.664 | 0.148 | -1.957 | -1.373 |
| **Reward x Effort** | 0.120 | 0.235 | -0.345 | 0.587 |
