# Supplementary material for: Dopamine and acetylcholine have distinct roles in delay- and effort-based decision-making in humans
Source: PLoS Biol. 2024 Jul 12;22(7):e3002714. doi: 10.1371/journal.pbio.3002714 (PMC11268711; doi:10.1371/journal.pbio.3002714)
Supplement: S5 Table — (DOCX) [file pbio.3002714.s017.docx]

**S5 Table.** Bayesian Generalized Linear Mixed Models of the Delay Discounting Task – Baseline Session; Regressing Choices (High-Cost vs. Low-Cost Option) on Predictors for Reward (High-Cost Option Reward), Delay (High-Cost Option Delay), and their Interaction Terms.

| **Parameter** | **Estimate** | **Est. Error** | **2.5%** | **97.5%** |
| --- | --- | --- | --- | --- |
| **(Intercept)** | 25.867 | 4.242 | 17.793 | 34.506 |
| **Reward** | 72.914 | 11.447 | 50.953 | 96.234 |
| **Delay** | -3.793 | 1.036 | -5.985 | -1.853 |
| **Reward x Delay** | -6.699 | 2.927 | -12.868 | -1.206 |
