# Supplementary material for: Dopamine and acetylcholine have distinct roles in delay- and effort-based decision-making in humans
Source: PLoS Biol. 2024 Jul 12;22(7):e3002714. doi: 10.1371/journal.pbio.3002714 (PMC11268711; doi:10.1371/journal.pbio.3002714)
Supplement: S6 Table — (DOCX) [file pbio.3002714.s018.docx]

**S6 Table.** Bayesian Linear Mixed Models of the Effort Discounting Task, Regressing Decision Times on Predictors for Drug, Reward (Difference between High-Cost vs. Low-Cost Reward Level), Effort (Difference between High-Cost vs. Low-Cost Effort Level), and their Interaction Terms.

| **Parameter** | **Estimate** | **Est. Error** | **2.5%** | **97.5%** |
| --- | --- | --- | --- | --- |
| **(Intercept)** | 7.047 | 0.038 | 6.973 | 7.124 |
| **Biperiden** | -0.009 | 0.027 | -0.062 | 0.045 |
| **Haloperidol** | 0.016 | 0.032 | -0.047 | 0.078 |
| **Reward** | -0.183 | 0.015 | -0.213 | -0.154 |
| **Effort** | 0.084 | 0.011 | 0.063 | 0.105 |
| **Biperiden x Reward** | 0.005 | 0.015 | -0.024 | 0.035 |
| **Haloperidol x Reward** | 0.036 | 0.014 | 0.009 | 0.063 |
| **Biperiden x Effort** | 0.002 | 0.012 | -0.022 | 0.025 |
| **Haloperidol x Effort** | -0.021 | 0.013 | -0.047 | 0.003 |
| **Reward x Effort** | 0.083 | 0.016 | 0.051 | 0.116 |
| **Biperiden x Reward x Effort** | -0.020 | 0.023 | -0.067 | 0.026 |
| **Haloperidol x Reward x Effort** | 0.000 | 0.023 | -0.045 | 0.047 |
