# Supplementary material for: Dopamine and acetylcholine have distinct roles in delay- and effort-based decision-making in humans
Source: PLoS Biol. 2024 Jul 12;22(7):e3002714. doi: 10.1371/journal.pbio.3002714 (PMC11268711; doi:10.1371/journal.pbio.3002714)
Supplement: S7 Table — (DOCX) [file pbio.3002714.s019.docx]

**S7 Table.** Bayesian Linear Mixed Models of the Delay Discounting Task, Regressing Decision Times on Predictors for Drug, Reward (High-Cost Option Reward), Delay (High-Cost Option Delay), and their Interaction Terms.

| **Parameter** | **Estimate** | **Est. Error** | **2.5%** | **97.5%** |
| --- | --- | --- | --- | --- |
| **(Intercept)** | 6.974 | 0.028 | 6.922 | 7.032 |
| **Biperiden** | 0.019 | 0.023 | -0.027 | 0.065 |
| **Haloperidol** | -0.077 | 0.023 | -0.123 | -0.031 |
| **Reward** | -0.168 | 0.011 | -0.190 | -0.145 |
| **Delay** | 0.036 | 0.008 | 0.020 | 0.053 |
| **Biperiden x Reward** | 0.002 | 0.010 | -0.017 | 0.022 |
| **Haloperidol x Reward** | 0.029 | 0.009 | 0.011 | 0.046 |
| **Biperiden x Delay** | -0.003 | 0.008 | -0.020 | 0.013 |
| **Haloperidol x Delay** | -0.020 | 0.008 | -0.036 | -0.003 |
| **Reward x Delay** | 0.005 | 0.011 | -0.017 | 0.027 |
| **Biperiden x Reward x Delay** | 0.013 | 0.016 | -0.019 | 0.044 |
| **Haloperidol x Reward x Delay** | -0.002 | 0.016 | -0.033 | 0.030 |
