# Supplementary material for: Dopamine and acetylcholine have distinct roles in delay- and effort-based decision-making in humans
Source: PLoS Biol. 2024 Jul 12;22(7):e3002714. doi: 10.1371/journal.pbio.3002714 (PMC11268711; doi:10.1371/journal.pbio.3002714)
Supplement: S8 Table — (DOCX) [file pbio.3002714.s020.docx]

**S8 Table.** Fixed effects from robust linear regression model with κ as dependent variable and questionnaire total scores, sex, and age as independent variable for the effort discounting task.

| **Variables** | **Parameter Estimates** | **Standard Error** | ***z*** | ***p*** |
| --- | --- | --- | --- | --- |
| **(Intercept)** | -0.059 | 0.051 | -1.157 | 0.252 |
| **Sex** | 0.010 | 0.016 | 0.661 | 0.511 |
| **Age** | 0.007 | 0.002 | 2.770 | **0.008** |
| **BIS-15** | -0.006 | 0.008 | -0.828 | 0.411 |
| **AES** | 0.006 | 0.009 | 0.709 | 0.481 |
| **BDI** | -0.006 | 0.011 | -0.584 | 0.562 |
