# Supplementary material for: Dopamine and acetylcholine have distinct roles in delay- and effort-based decision-making in humans
Source: PLoS Biol. 2024 Jul 12;22(7):e3002714. doi: 10.1371/journal.pbio.3002714 (PMC11268711; doi:10.1371/journal.pbio.3002714)
Supplement: S9 Table — (DOCX) [file pbio.3002714.s021.docx]

**S9 Table.** Fixed effects from robust linear regression model with κ as dependent variable and questionnaire total scores, sex, and age as independent variable for the delay discounting task.

| **Variables** | **Parameter Estimates** | **Standard Error** | ***z*** | ***p*** |
| --- | --- | --- | --- | --- |
| **(Intercept)** | -4.621 | 1.645 | -2.808 | **0.007** |
| **Sex** | -0.510 | 0.531 | -0.962 | 0.340 |
| **Age** | 0.011 | 0.076 | 0.146 | 0.885 |
| **BIS-15** | -0.197 | 0.282 | -0.700 | 0.487 |
| **AES** | 0.458 | 0.312 | 1.471 | 0.147 |
| **BDI** | -0.023 | 0.217 | -0.108 | 0.915 |
