# Supplementary material for: Dopamine and acetylcholine have distinct roles in delay- and effort-based decision-making in humans
Source: PLoS Biol. 2024 Jul 12;22(7):e3002714. doi: 10.1371/journal.pbio.3002714 (PMC11268711; doi:10.1371/journal.pbio.3002714)
Supplement: S10 Table — (DOCX) [file pbio.3002714.s022.docx]

**S10 Table.** Fixed effects from robust linear regression model with κ as dependent variable and questionnaire subscales as independent variable for the effort discounting task.

| **Variables** | **Parameter Estimates** | **Standard Error** | ***z*** | ***p*** |
| --- | --- | --- | --- | --- |
| **(Intercept)** | 0.098 | 0.007 | 13.088 | **< 0.001** |
| **BIS-15 attentional** | 0.005 | 0.011 | 0.497 | 0.6212 |
| **BIS-15 motor** | -0.025 | 0.013 | -1.863 | 0.0678 |
| **BIS-15 non-planning** | 0.010 | 0.007 | 1.475 | 0.1459 |
| **AES - Apathy** | 0.016 | 0.009 | 1.781 | 0.0804 |
| **AES - Disinterest** | -0.008 | 0.007 | -1.083 | 0.2835 |
| **AES – Social Withdrawal** | 0.007 | 0.011 | 0.579 | 0.5650 |
