# Supplementary material for: Dopamine and acetylcholine have distinct roles in delay- and effort-based decision-making in humans
Source: PLoS Biol. 2024 Jul 12;22(7):e3002714. doi: 10.1371/journal.pbio.3002714 (PMC11268711; doi:10.1371/journal.pbio.3002714)
Supplement: S11 Table — (DOCX) [file pbio.3002714.s023.docx]

**S11 Table.** Fixed effects from robust linear regression model with κ as dependent variable and questionnaire subscales as independent variable for the delay discounting task.

| **Variables** | **Parameter Estimates** | **Standard Error** | ***z*** | ***p*** |
| --- | --- | --- | --- | --- |
| **(Intercept)** | -4.728 | 0.228 | -20.717 | **< 0.001** |
| **BIS-15 attentional** | -0.605 | 0.336 | -1.801 | 0.0771 |
| **BIS-15 motor** | 0.039 | 0.345 | 0.114 | 0.9100 |
| **BIS-15 non-planning** | 0.224 | 0.286 | 0.783 | 0.4372 |
| **AES - Apathy** | 0.311 | 0.294 | 1.056 | 0.2957 |
| **AES - Disinterest** | 0.432 | 0.262 | 1.647 | 0.1054 |
| **AES – Social Withdrawal** | -0.252 | 0.232 | -1.086 | 0.2824 |
