# Supplementary material for: Dopamine and acetylcholine have distinct roles in delay- and effort-based decision-making in humans
Source: PLoS Biol. 2024 Jul 12;22(7):e3002714. doi: 10.1371/journal.pbio.3002714 (PMC11268711; doi:10.1371/journal.pbio.3002714)
Supplement: S12 Table — (DOCX) [file pbio.3002714.s024.docx]

**S12 Table.** Bayesian Generalized Linear Mixed Models of the Effort Discounting Task – Fatigue Effects; Regressing Choices (High-Cost vs. Low-Cost Option) on Predictors for Drug, Reward (Difference between High-Cost vs. Low-Cost Reward Level), Effort (Difference between High-Cost vs. Low-Cost Effort Level), and their Interaction Terms, as well as Trial Number and two-way Trial number x Drug interactions.

| **Parameter** | **Estimate** | **Est. Error** | **2.5%** | **97.5%** |
| --- | --- | --- | --- | --- |
| **(Intercept)** | 2.602 | 0.224 | 2.169 | 3.056 |
| **Biperiden** | 0.606 | 0.210 | 0.206 | 1.030 |
| **Haloperidol** | -0.475 | 0.216 | -0.903 | -0.045 |
| **Reward** | 3.516 | 0.256 | 3.037 | 4.049 |
| **Delay** | -1.682 | 0.122 | -1.919 | -1.438 |
| **Trial Number** | -0.605 | 0.073 | -0.747 | -0.462 |
| **Biperiden x Reward** | 0.792 | 0.299 | 0.218 | 1.398 |
| **Haloperidol x Reward** | -0.196 | 0.272 | -0.718 | 0.348 |
| **Biperiden x Delay** | 0.012 | 0.157 | -0.294 | 0.328 |
| **Haloperidol x Delay** | 0.024 | 0.124 | -0.221 | 0.274 |
| **Biperiden x Trial Number** | 0.142 | 0.107 | -0.068 | 0.350 |
| **Haloperidol x Trial Number** | -0.431 | 0.101 | -0.632 | -0.232 |
| **Reward x Delay** | 0.132 | 0.186 | -0.241 | 0.490 |
| **Biperiden x Reward x Delay** | 0.117 | 0.298 | -0.480 | 0.676 |
| **Haloperidol x Reward x Delay** | -0.262 | 0.248 | -0.740 | 0.238 |
