# Supplementary material for: Dopamine and acetylcholine have distinct roles in delay- and effort-based decision-making in humans
Source: PLoS Biol. 2024 Jul 12;22(7):e3002714. doi: 10.1371/journal.pbio.3002714 (PMC11268711; doi:10.1371/journal.pbio.3002714)
Supplement: S13 Table — (DOCX) [file pbio.3002714.s025.docx]

**S13 Table.** Bayesian Generalized Linear Mixed Models of the Delay Discounting Task – Fatigue Effects; Regressing Choices (High-Cost vs. Low-Cost Option) on Predictors for Drug, Reward (High-Cost Option Reward), Delay (High-Cost Option Delay), and their Interaction Terms, as well as Trial Number and two-way Trial number x Drug interactions.

| **Parameter** | **Estimate** | **Est. Error** | **2.5%** | **97.5%** |
| --- | --- | --- | --- | --- |
| **(Intercept)** | 20.230 | 2.637 | 15.137 | 25.461 |
| **Biperiden** | -1.318 | 1.235 | -4.043 | 0.695 |
| **Haloperidol** | -0.019 | 1.080 | -2.477 | 1.921 |
| **Reward** | 57.451 | 7.123 | 43.829 | 71.553 |
| **Delay** | -2.295 | 0.537 | -3.341 | -1.240 |
| **Trial Number** | -0.204 | 0.064 | -0.326 | -0.079 |
| **Biperiden x Reward** | -3.878 | 3.407 | -11.446 | 1.564 |
| **Haloperidol x Reward** | -1.219 | 2.927 | -7.948 | 4.017 |
| **Biperiden x Delay** | 0.792 | 0.490 | -0.106 | 1.843 |
| **Haloperidol x Delay** | 1.338 | 0.557 | 0.243 | 2.481 |
| **Biperiden x Trial Number** | 0.146 | 0.088 | -0.028 | 0.320 |
| **Haloperidol x Trial Number** | -0.003 | 0.087 | -0.173 | 0.163 |
| **Reward x Delay** | -2.808 | 1.409 | -5.618 | -0.059 |
| **Biperiden x Reward x Delay** | 1.086 | 1.363 | -1.365 | 4.061 |
| **Haloperidol x Reward x Delay** | 2.369 | 1.554 | -0.687 | 5.574 |
