# Supplementary material for: Dopamine and acetylcholine have distinct roles in delay- and effort-based decision-making in humans
Source: PLoS Biol. 2024 Jul 12;22(7):e3002714. doi: 10.1371/journal.pbio.3002714 (PMC11268711; doi:10.1371/journal.pbio.3002714)
Supplement: S14 Table — (DOCX) [file pbio.3002714.s026.docx]

**S14 Table.** Bayesian Generalized Linear Mixed Models of the Effort Discounting Task – Session Effects; Regressing Choices (High-Cost vs. Low-Cost Option) on Predictors for Drug, Reward (Difference between High-Cost vs. Low-Cost Reward Level), Effort (Difference between High-Cost vs. Low-Cost Effort Level), and their Interaction Terms, as well as Session and two-way Session x Drug interactions.

| **Parameter** | **Estimate** | **Est. Error** | **2.5%** | **97.5%** |
| --- | --- | --- | --- | --- |
| **(Intercept)** | 2.542 | 0.213 | 2.133 | 2.966 |
| **Biperiden** | 0.594 | 0.199 | 0.217 | 1.007 |
| **Haloperidol** | -0.542 | 0.193 | -0.924 | -0.176 |
| **Reward** | 3.411 | 0.247 | 2.944 | 3.916 |
| **Delay** | -1.648 | 0.120 | -1.883 | -1.409 |
| **Session** | 0.488 | 0.183 | 0.125 | 0.852 |
| **Biperiden x Reward** | 0.783 | 0.288 | 0.253 | 1.373 |
| **Haloperidol x Reward** | -0.284 | 0.250 | -0.776 | 0.207 |
| **Biperiden x Delay** | -0.018 | 0.149 | -0.308 | 0.279 |
| **Haloperidol x Delay** | 0.092 | 0.121 | -0.142 | 0.326 |
| **Biperiden x Session** | -0.158 | 0.305 | -0.758 | 0.445 |
| **Haloperidol x Session** | -0.426 | 0.293 | -1.018 | 0.132 |
| **Reward x Delay** | 0.148 | 0.182 | -0.216 | 0.499 |
| **Biperiden x Reward x Delay** | 0.090 | 0.290 | -0.486 | 0.642 |
| **Haloperidol x Reward x Delay** | -0.222 | 0.237 | -0.685 | 0.241 |
