# Supplementary material for: Dopamine and acetylcholine have distinct roles in delay- and effort-based decision-making in humans
Source: PLoS Biol. 2024 Jul 12;22(7):e3002714. doi: 10.1371/journal.pbio.3002714 (PMC11268711; doi:10.1371/journal.pbio.3002714)
Supplement: S15 Table — (DOCX) [file pbio.3002714.s027.docx]

**S15 Table.** Bayesian Generalized Linear Mixed Models of the Delay Discounting Task – Session Effects; Regressing Choices (High-Cost vs. Low-Cost Option) on Predictors for Drug, Reward (High-Cost Option Reward), Delay (High-Cost Option Delay), and their Interaction Terms, as well as Session and two-way Session x Drug interactions.

| **Parameter** | **Estimate** | **Est. Error** | **2.5%** | **97.5%** |
| --- | --- | --- | --- | --- |
| **(Intercept)** | 20.491 | 2.751 | 15.007 | 25.963 |
| **Biperiden** | -1.355 | 1.260 | -4.100 | 0.616 |
| **Haloperidol** | 0.024 | 1.121 | -2.543 | 2.007 |
| **Reward** | 58.132 | 7.390 | 43.331 | 72.868 |
| **Delay** | -2.286 | 0.539 | -3.353 | -1.230 |
| **Session** | 0.400 | 0.242 | -0.062 | 0.871 |
| **Biperiden x Reward** | -3.924 | 3.464 | -11.646 | 1.420 |
| **Haloperidol x Reward** | -1.081 | 3.032 | -8.141 | 4.302 |
| **Biperiden x Delay** | 0.787 | 0.487 | -0.087 | 1.843 |
| **Haloperidol x Delay** | 1.347 | 0.567 | 0.219 | 2.495 |
| **Biperiden x Session** | -0.064 | 0.375 | -0.807 | 0.657 |
| **Haloperidol x Session** | -0.315 | 0.370 | -1.044 | 0.392 |
| **Reward x Delay** | 1.088 | 1.349 | -1.314 | 4.003 |
| **Biperiden x Reward x Delay** | 2.430 | 1.585 | -0.660 | 5.601 |
| **Haloperidol x Reward x Delay** | -2.809 | 1.429 | -5.678 | 0.008 |
