# Supplementary material for: Dopamine and acetylcholine have distinct roles in delay- and effort-based decision-making in humans
Source: PLoS Biol. 2024 Jul 12;22(7):e3002714. doi: 10.1371/journal.pbio.3002714 (PMC11268711; doi:10.1371/journal.pbio.3002714)
Supplement: Attachment — Submitted filename: Response_Letter_Erfanian_Abdoust.pdf [file pbio.3002714.s028.pdf]

We would like to thank the reviewers very much for their thoughtful and constructive feedback. Their comments have helped us to a substantially improved manuscript. Specifically, we have revised the introduction and discussion to avoid overgeneralizations regarding the roles of dopamine and acetylcholine in cost-benefit decision making, ensuring that our interpretations are supported by our specific findings. We now also characterize the relationship between both neurotransmitter systems more cautiously, limiting contrasting effects to specific components of effort-based behaviour, rather than implying a fully antagonistic relationship. Further, we have incorporated additional analyses to control for potential confounding factors raised by the reviewers. Finally, we have expanded the methods section to provide a more comprehensive explanation of our analysis approach, including a more detailed description of the computational models, including model selection and parameter selection.

We reproduce each of the reviewers' comments below in italics before our response to each point. Changes made to the manuscript are in blue, both here and in the manuscript. In the tracked version of the manuscript, sections that are removed from the manuscript are highlighted by being strikethrough. When we refer to specific lines or sections in the manuscript, we refer to the tracked versions of the document.

Reviewer #1:

"*This manuscript asks the interesting question on the role of dopamine and acetylcholine in delay and effort-based decision making. While there is already several work on the role of dopamine in for example effort-based decision making, further examination of the role of dopamine in delay discounting and especially of acetylcholine in decision making more broadly seems relevant for a better understanding of the role of these neurotransmitters and their potential interplay. The authors report several analyses and control analyses using state-of-the-art analytical approaches and discuss their findings in relation to previous work as well as mentioning some limitations of their study. While well written in general, I believe the specific contribution of this study beyond previous work could be mapped out a bit more clearly in the introduction, while the authors should also be cautious and nuanced in their interpretation and generalisation of their findings. In addition, a few methodological details and some further potential limitations of their study due to the experimental design could be included. Specific comments and suggestions are detailed below."*

We thank you for your thoughtful comments and for recognizing the potential value of our study. In response to your feedback, we have made several revisions to enhance clarity of the manuscript and to address potential limitations regarding the experimental design.

R1.1) "*At several points in the manuscript, such as the abstract, discussion etc., the authors talk about a domain-general role of dopamine and a domain-specific role of acetylcholine. Given that only two types of costs (effort and delay) were tested here in only two different tasks, this conclusion might be a bit too broad. Furthermore, given the main effects and interaction effects found in decisions, reaction times and stochasticity, I believe the conclusion drawn - and the statement made - that the acetylcholine receptor antagonist produced opposite effects as the dopamine receptor antagonist goes a bit too far (Also the fact that no correlations were found in the across-task relationship analyses might indicate that the effects are not fully opposing). Instead of drawing such broad conclusions, it may be better to stick to the actual findings when summarizing and interpreting them (and only briefly speculate about domain-general versus domain-specific as well as opposing effects).*"

We fully agree – we also think it is important not to overstate/overgeneralize our research findings. Indeed, as you pointed out, the terms "domain-general" and "domain-specific" were potentially misleading and overly broad, given our focus on two specific types of cost.
We have thoroughly revised the manuscript (abstract, lines 33 - 35; introduction, lines 97 - 102; and discussion, lines 601 - 603) to avoid these terms, using them now only in the discussion section for speculation about potential broader effects.

Abstract:

> Together, our data suggest that dopamine, acting at D2 receptors, modulates both effort and delay discounting, while acetylcholine, acting at M1 receptors, appears to exert a more specific influence on effort discounting only.

Introduction:

> Second, we aimed to assess the contribution of acetylcholine to cost-benefit decision making and conceptually contrast it with the effects of dopamine. In doing so, we investigated whether any effects of muscarinic or dopaminergic receptor antagonism would modulate the computation of both time and effort costs or have a more specific effect on one cost dimension.

Discussion:

> Our findings indicate that, while D2 receptor activity plays a role in integrating both delay and effort costs, acetylcholine, acting at M1 receptors, may have a more specific role in effort processing.

Additionally, we have now rewritten the abstract (lines 26 - 30), introduction (lines 113 - 116), results (lines 320 - 322), and discussion (lines 500 - 511) section regarding opposing mechanistic effects between haloperidol and biperiden. We now explicitly link these opposing effects to specific features of effort-based decision making: the modulation of the general willingness to exert effort (as shown in the regression model) and the effort discounting parameter (as shown in the computational modelling analysis). However, we acknowledge that some other components of effort-based decision making (such as decision times and drug effects on reward sensitivity) exhibited differential, rather than directly opposing effects. The revised sections read as follows:

Abstract:

> We found opposing effects of dopamine D2 (haloperidol) and acetylcholine M1 receptor (biperiden) antagonism on specific components of effort-based decision making in healthy humans: haloperidol decreased, whereas biperiden increased the willingness to exert physical effort.

Introduction:

> In brief, we found opposing effects of haloperidol and biperiden only on specific components of effort-based choices. Specifically, haloperidol reduced the willingness to invest physical effort, whereas biperiden increased it.

Results:

> This implies that contrasting effects of dopaminergic and cholinergic manipulations may reflect computationally-specific, rather than universal effects.

Discussion:

> Indeed, we observed opposing effects of dopaminergic and cholinergic manipulations within specific components of effort-, but not delay-based decision making. Specifically, we found evidence for biperiden increasing the willingness to invest effort for rewards and, in line with this, decreasing effort discounting. In contrast, haloperidol reduced the general willingness to invest effort and increased effort discounting. However, we also observed drug effects that were not in opposite directions between biperiden and haloperidol. For example, biperiden increased reward sensitivity, while haloperidol had no credible effect on the impact of rewards on effort-based choices. Furthermore, haloperidol modulated decision times in both experimental tasks, whereas biperiden did not affect them. These findings suggest partially opposing

effects between both neurotransmitters, mostly evident within specific components of effort-based choices.

R1.2a) "*Somewhat relatedly, in their final conclusion (page 19) the authors suggest a reciprocal relationship between cholinergic and dopaminergic activity. Given that the two neurotransmitters were manipulated in separate sessions, such a relationship seems speculative.*

Thank you for pointing this out. We fully agree that the present set of experiments was not designed to investigate reciprocal cholinergic-dopaminergic relationships. Please note however that this was not the purpose of our manuscript. Such reciprocal relationships at the level of the striatum have been documented extensively in the literature (e.g., Gerber et al. 2001, *PNAS*; Stanhope et al. 2001, *J Pharm Exp Ther*). Instead, it was on the basis of these findings that we had hypothesized that cholinergic (M1) receptors should have an effect on cost-benefit decision making that is opposite to that of dopamine. We apologize for the confusion. To clarify this point, we have now reworded the paragraph (discussion, lines 599 - 603) you are referring to, which now reads as follows (see also #R1.1da):

> This suggests that in the context of human cost-benefit decision making, the previously reported reciprocal relationship between both neurotransmitters may be limited only to specific components of behaviour. Our findings indicate that, while D2 receptor activity plays a role in integrating both delay and effort costs, acetylcholine, acting at M1 receptors, may have a more specific role in effort processing.

R1.2b) *"Furthermore, the authors mention a domain-general role of dopamine in integrating proximity and effort costs. Again, since proximity and effort costs were manipulated in separate tasks, such an integration cannot certainly be concluded from this experiment, strictly speaking. I would prefer it if this was formulated more cautiously."*

As you correctly mentioned in #R1.1, the term "domain-general" is overinterpreted and we avoid using the term now. Further, we acknowledge that the word *proximity* in this context may be misleading.

Regarding the role of psychological proximity in our study, it is important to clarify that we only introduced the term in the discussion to relate our findings to a theoretical framework described by Soutschek et al. (2023, *Biol Psychiatry Glob Open Sci*). This theory suggests that dopamine plays a dual role in (i) biasing choices towards options with a psychological proximity advantage and (ii) modulating the computation of acceptable costs in return for

rewards. This theory aligns with our findings from the behavioural experiment. However, we acknowledge that this idea is highly speculative and requires further investigation. As we did not explicitly control for psychological proximity in our task design, any conclusions about the integration of proximity costs by dopamine cannot be made. We have revised the relevant section to be less definitive and more speculative in tone (lines 433 - 451).

> In other words, this theoretical framework proposes that dopamine may play a dual role in (i) promoting choices towards options with a psychological proximity advantage and (ii) weighing reward magnitudes against associated costs. Our findings of decreased delay discounting under haloperidol align with this theory, potentially suggesting a diminished preference for more proximal options when D2-receptor activity is reduced, resulting in increased endurance for higher (time) costs. On the other hand, the reduction of dopaminergic neurotransmission had the opposite effect on effort discounting, leading to a diminished willingness to bear higher (effort) costs. This apparently conflicting finding between effort and time costs may be explained by the fact that neither the low-effort nor the high-effort option had a pronounced psychological proximity advantage. Thus, in this specific context of cost-benefit decision making, the proximity factor appears to be less relevant, and dopamine instead may promote behaviour aimed at maximizing rewards simply by weighing rewards against associated costs. Importantly, our experimental paradigm did not directly manipulate psychological proximity, nor did it distinguish between proximity effects and the impact of delay. Therefore, specific effects of dopamine on psychological proximity in human delay discounting remain highly speculative and warrant further investigation with experimental paradigms specifically tailored to this question.

Moreover, we have revised the corresponding conclusion (discussion, lines 595 - 603):

> Further, we demonstrate that the administration of biperiden, a muscarinic M1 receptor antagonist, had contrasting effects to those of dopaminergic D2 receptor antagonism in the general willingness to choose high-cost options and the effort discounting parameter. This suggests that in the context of human cost-benefit decision making, the previously reported reciprocal relationship between both neurotransmitters may be limited only to specific components of behaviour. Our findings indicate that, while D2 receptor activity plays a role in integrating both delay and effort costs, acetylcholine, acting at M1 receptors, may have a more specific role in effort processing.

R1.3) “*The introduction could be extended by further detailing previous results on the effects of dopamine on effort sensitivity and reward sensitivity and by further detailing previous findings on the role of dopamine in delay discounting. In addition, previous research on potential related roles of acetylcholine could be explicitly referred to in the introduction. Providing a bit more detail on previous related research may help the reader to better understand the contributions and hypothesis of this manuscript.*“

Thank you for this suggestion. We have extended the introduction to include more details on the role of dopamine in both effort- and delay-based decision making. Additionally, we have included more information on the evidence suggesting acetylcholine’s involvement in these decisions. We believe that this will further clarify the context and rationale of our study. The revised introduction now reads as follows (see lines 55 - 86):

> Pharmacological studies consistently demonstrate that agents that enhance dopaminergic transmission in humans increase reward sensitivity and decrease effort sensitivity, while studies directly investigating the effects of selective D2-receptor antagonism in healthy humans are rare (Bogdanov et al., 2022; Chong et al., 2023; Le Bouc et al., 2016). For delay discounting, the existing literature is somewhat more mixed. Some studies suggest that increased dopamine transmission decreases delay discounting (de Wit et al., 2002; Floresco et al., 2008; Kayser et al., 2012), whereas others have found either no effect (Acheson & de Wit, 2008; Hamidovic et al., 2008) or an increase in delay discounting (Pine et al., 2010). Notably, more recent evidence in humans indicates decreased delay discounting after blockade of D2 receptors (Soutschek & Tobler, 2023; Wagner et al., 2020). […] In line with this, animal studies indicate that muscarinic agonists induce behavioural changes in effort-based choices that are similar to those produced by dopamine antagonists (Hailwood et al., 2019; Nunes et al., 2013), underscoring the potential interplay between dopamine and acetylcholine in modulating cost-benefit decision making. Furthermore, research in animal models points to a role for acetylcholine in modulating delay-based choices through both muscarinic and nicotinic receptors, albeit with inconsistent findings (Dallery & Locey, 2005; Kolokotroni et al., 2011; Mendez et al., 2012; Ozga & Anderson, 2018). Importantly, studies directly investigating these effects in human decision making are lacking.

R1.4) “*Since participants were required to exert the chosen effort on every trial (albeit briefly), it seems plausible that fatigue could have developed in the effort discounting task (see e.g. Müller et al., 2021, Nature Communications; Matthews et al., 2023, Cognition) and might have*

affected the results and potentially contributed to the differences observed between tasks. If not modelled explicitly, this should at least be mentioned in the discussion."

This is an important point. To address this, we re-ran the GLMMs with the following additional regressors: *trial number*, to account for the main effect of fatigue over the course of the experiment, and the *trial number x drug* interaction, to test whether the drugs differentially affected how fatigue might influence choices. This analysis revealed a main effect of trial number (HDI_{Mean} = -0.60, $HDI_{95\%}$ = [-0.75; -0.46]), suggesting a general fatigue effect throughout the task. Importantly, we also found a significant interaction between trial number and haloperidol (HDI_{Mean} = -0.43, $HDI_{95\%}$ = [-0.63; -0.23]). This indicates that under haloperidol, the fatigue effect during the experimental session was more pronounced compared to placebo. Importantly, adding the new predictors did not change our main findings (main effect of both drugs in the effort discounting task; credible delay x haloperidol interaction in the delay discounting task; credible main effects of all reward and cost manipulations), indicating a dual role of haloperidol in reducing the willingness to invest effort and exacerbating the fatigue effect throughout the task. We have now added this control analysis to the results section to address the potential role of fatigue in both the effort and delay discounting task in the manuscript (results, lines 186 - 202):

> Having established distinct task-specific drug effects on participants' choice behaviour, we next investigated potential confounding order and fatigue effects using separate GLMMs for effort- and delay-based decision making. These models mirrored the previously described GLMMs but included additional predictors to test for fatigue and session effects and their modulation by drugs.
>
> To test for possible fatigue effects, we added trial number as well as the two-way interaction with drug as additional predictors. This analysis revealed a credible main effect of trial number for both the effort and delay discounting task (trial number effect on effort discounting: HDI_{Mean} = -0.60, $HDI_{95\%}$ = [-0.75; -0.46]; trial number effect on delay discounting: HDI_{Mean} = -0.20, $HDI_{95\%}$ = [-0.33; -0.08]), suggesting that the tendency to choose high-cost options, irrespective of cost type, decreased over the course of the experiment. Notably, we found a credible interaction effect between trial number and haloperidol in the effort discounting task (HDI_{Mean} = -0.43, $HDI_{95\%}$ = [-0.63; -0.23]), indicating that the fatigue effect was more pronounced under haloperidol compared to placebo. Importantly, even after accounting for these fatigue-related effects, the main effect of haloperidol on effort discounting remained credible (HDI_{Mean} = -0.48, $HDI_{95\%}$ = [-0.90; -0.05]) (Supplementary Table 12 and 13).

Please note that both Müller et al. and Matthews et al. used computational models to distinguish different states of fatigue. This is beyond the capabilities of our regression models. We initially explored the inclusion of additional drug-sensitive parameters for both fatigue states into our computational model. However, this led to severe convergence issues and unreliable parameter estimates, likely due to the model becoming overparameterized and due to differences in our task design. This also aligns with the broader literature on hierarchical modelling of effort discounting, as similar discounting frameworks (i.e., those that analyse condition effects on free parameters), commonly use only the κ parameter in their discounting models. We have incorporated this limitation in the revised discussion section (lines 551 - 564).

> Third, as participants were required to exert physical effort on each trial, fatigue effects could have developed in the effort discounting task. While our additional analysis confirmed a general fatigue effect and a dual role of haloperidol in both reducing the overall propensity to invest effort and in exacerbating the fatigue effect throughout the task, we acknowledge that recent studies revealed the existence of distinct states of fatigue (Matthews et al., 2023; Müller et al., 2021). Importantly, this was discovered by using task paradigms and computational models that were designed to distinguish between these different types of fatigue. Additionally, it is important to consider that potential motor effects, particularly in the context of dopaminergic manipulations, could also affect effort discounting behaviour. Future studies could extend our approach by incorporating measurements of force pulses to investigate potential motor-related effects and apply task designs and computational models specifically tailored to capture how drug manipulations might affect different forms of fatigue.

R1.5) "*Relatedly, could the authors clarify why the order of the tasks was fixed, and discuss whether the observed effects could also be confounded by a potential order effect (potentially due to fatigue, or boredom, satiety, etc.)?*"

This is something we had actually discussed in great detail while planning the study. While there are certainly good reasons to balance task order, we opted to keep the order fixed, mostly for the following two reasons: First, we wanted to make sure that both tasks were administered at the same time relative to drug administration to reduce across-subject variability in drug levels, yielding more consistent drug levels at the time of testing. Second, we wanted to administer the effort discounting task first due to its greater physical demand. This aimed to minimize the influence of fatigue on the physically more challenging effort task.

Our within-subjects design allows us to compare each participant's behaviour under drug directly to their own behaviour under placebo. We believe this helps to mitigate the impact of task order effects, as any systematic influence of fatigue or boredom would likely be present under all conditions. However, we acknowledge that possible effects of fatigue, boredom, or satiety could still contribute to different effects of the drugs in the delay discounting task. This again underscores a limitation in directly comparing effort- and delay-based decision making, as these aspects were tested at different times during the experiment and under different phases of the drug manipulation. We have now incorporated a new paragraph into the limitations section, where we explicitly discuss this issue along with other factors that preclude direct comparisons (discussion, lines 564 - 577):

> Lastly, it is important to note that several factors preclude a direct quantitative comparison between the delay and effort discounting tasks. These include the distinct nature of rewards and costs (hypothetical vs. real), varying reward magnitudes in both tasks (large vs. small), and differences in task structure (fixed vs. variable alternative option). Additionally, both tasks were consistently performed in a fixed order within and between participants (effort followed by delay). While this ensured consistent drug levels within each task, it is potentially introducing task order effects and possibly leading to different drug concentrations between tasks. These methodological differences limit our ability to directly compare drug effects across the two cost domains. Future research would benefit from applying experimental paradigms that manipulate both delay and effort costs within the same task, allowing a more controlled and direct comparison of how pharmacological manipulations differentially influence sensitivity to these distinct cost types.

R1.6) "*A few things regarding the design and methodology could be clarified and described in more detail. For example*"

R1.6a) "*Could the authors clarify why a fixed alternative was used in the delay discounting task but not in the effort discounting task, which also makes the dependent variables in the regression analyses somewhat hard to compare between the tasks?*"

It is true that the design differences make the regression results between the tasks less comparable.

However, in the effort discounting task, we deviated from the standard approach, which is either a two-alternative choice task with a fixed alternative or an accept/reject paradigm, for the following reasons:

First, it is more ecologically valid to apply a two-alternative choice paradigm with varying options, as this more closely mirrors real-world decision scenarios, where individuals typically have to choose between multiple options with varying levels in both cost and reward dimension.
Second, in our own prior work with a clinical population, we had used the accept/reject paradigm of the effort discounting task (comparable with a fixed alternative design). This showed that many participants exclusively, or almost exclusively, chose the high-reward/high-effort option. This resulted in a substantial number of participants that did not show any discounting behaviour at all (Erfanian Abdoust et al., 2024, *Brain Cogn*).

To address this issue, prior to starting our experiment, we had performed a set of pilot studies with healthy participants (N = 10) without pharmacological intervention. We used two-alternative choice paradigms with varying rewards and costs on both options for both the effort and the delay discounting. These pilot studies demonstrated the following:

A) All participants exhibited effort discounting behaviour in the effort discounting task (probability of choosing the high-cost option: M = 0.75; range = 0.55 – 0.94).
B) Surprisingly, compared to prior studies using the fixed-alternative design, we observed a higher number of participants showing very little to no delay discounting behaviour (probability of choosing the high-cost option: M = 0.75; range = 0.25 – 1.00). Specifically, 3 out of 10 participants chose the high-reward/high-delay option in more than 95% of trials, with none of the same participants showing this strong preference in the effort discounting task. Fig. R1 shows a comparison between choice behaviour in the pilot study and main experiment.

Based on these finding, we decided to use the two-alternative version with varying options for the effort discounting task. However, we opted for the established, fixed-alternative version for the delay discounting task. Given that the latter is also the standard in the literature, this allows comparability of our findings with the extensive existing literature. That said, we of course acknowledge that the difference in task structure represents a further potential limitation regarding direct comparisons between both tasks.

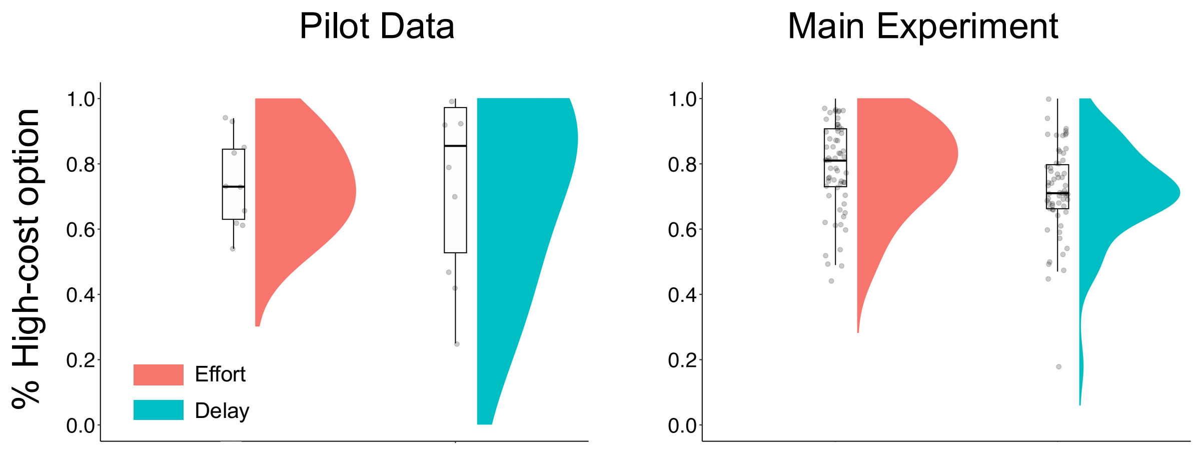

Fig.R1 Comparison of Choice Behaviour in the Pilot and Main Experiment. The figure shows the overall proportion of choosing the high-cost option in the pilot data (left panel) and in the main experiment (right panel). Note that the pilot data reflects two-alternative versions for both tasks. The delay discounting task in the pilot study revealed high choice variability and, importantly, in some participants, a strong bias towards high-cost options compared to the fixed-alternative version used in the main experiment

As mentioned in comment #R1.6a, we have now elaborated our discussion of these methodological differences in the limitations section (discussion, lines 564 - 577).

> Lastly, it is important to note that several factors preclude a direct quantitative comparison between the delay and effort discounting tasks. These include the distinct nature of rewards and costs (hypothetical vs. real), varying reward magnitudes in both tasks (large vs. small), and differences in task structure (fixed vs. variable alternative option). Additionally, both tasks were consistently performed in a fixed order within and between participants (effort followed by delay). While this ensured consistent drug levels within each task, it is potentially introducing task order effects and possibly leading to different drug concentrations between tasks. These methodological differences limit our ability to directly compare drug effects across the two cost domains. Future research would benefit from applying experimental paradigms that manipulate both delay and effort costs within the same task, allowing a more controlled and direct comparison of how pharmacological manipulations differentially influence sensitivity to these distinct cost types.

R1.6b) "*How much time did participants have to make their decision in each task?*"

Participants were given unlimited time to make their decisions in both tasks. This information has been added to the methods section for clarity (methods, lines 695 - 697 and 724 - 726).

Effort Discounting Task:

> During each trial, they had to choose between a high-reward/high-effort (high-cost) and a low-reward/low-effort (low-cost) offer. Participants had unlimited time for their decision.

Delay Discounting Task:

> In this task, they were presented with a similar choice paradigm, but this time between a high-reward/high-delay (high-cost) and a low-reward/low-delay (low-cost) option on each trial, again without any time limit.

R1.6c) "*Did every participant see the same trials in the same sequence?*"

The trial order in the effort discounting task was fully randomized for each participant and experimental session. For the delay discounting task, we used a pseudorandomized trial order, counterbalanced across experimental sessions but not across participants. We have added this information to the methods section (lines 713 - 714 and 734 - 736).

Effort Discounting Task:

> Each participant completed a total of 125 trials, divided into 5 blocks. The trial structure was fully randomized for each participant and experimental session.

Delay Discounting Task:

> Participants completed two blocks, with each block containing all unique combinations, resulting in a total of 210 trials. We used a pseudorandomized trial order, counterbalanced across experimental sessions but not across participants.

R1.6da) "*Page 31, line 731: Why was here, in contrast to the other analyses, no Bayesian framework used?*"

While our primary analyses indeed used a Bayesian framework, we had chosen frequentist methods for the control analyses as we had reasoned these analyses were much less sophisticated and didn't require the same computational advantages of Bayesian methods. However, in light of your feedback, we now use a Bayesian framework for the control analysis to ensure methodological coherence. These Bayesian analyses confirmed our previous findings: biperiden credibly reduced heart rate and systolic blood pressure at T_1 and at T_2, while both drugs reduced alertness at T_2. Moreover, the Bayesian regression models did not

show any significant drug effects on Trail-Making performance, MVC, or effort rating, confirming our previous findings. The corresponding methods (lines 904 - 906), supplementary methods (lines 285 - 294), and supplementary results (lines 82 - 91) sections now read as follows:

Methods:

> The effects of the drugs on mood (alertness, calmness, and contentedness) and physiological measures (heart rate, systolic, and diastolic blood pressure) were analysed using Bayesian Linear Mixed Models.

Supplementary methods:

> To analyse the effects of the drugs on mood ratings, physiological measures, trail-making test response times, MVC, and subjective effort perception ratings, we applied Bayesian Linear Mixed Models. […] For parameter estimation, we used non-informative priors (the brms default) and ran four chains with 3000 samples (1000 samples for warmup).

Supplementary results:

> Bayesian Linear Mixed Models revealed that biperiden administration led to a reduction in heart rate and systolic blood pressure at both T_1 (heart rate: HDI_{Mean} = -6.82, $HDI_{95\%}$ = [-10.74; - 2.97]; systolic blood pressure: HDI_{Mean} = -3.91, $HDI_{95\%}$ = [-7.74; -0.13]) and T_2 (heart rate: HDI_{Mean} = -7.88, $HDI_{95\%}$ = [-11.83; -3.89]; systolic blood pressure: HDI_{Mean} = -4.80, $HDI_{95\%}$ = [-8.74; -0.95]), with no significant impact on diastolic blood pressure (Supplementary Fig. 5). Moreover, subjective ratings of alertness were significantly lower under both haloperidol and biperiden at T_2 (biperiden: HDI_{Mean} = -0.15, $HDI_{95\%}$ = [-0.26; -0.05]; haloperidol: HDI_{Mean} = -0.15, $HDI_{95\%}$ = [-0.25; -0.04], Supplementary Fig. 6). Notably, we did not find significant drug effects on the response times in the trail-making task A, the subjective rating of effort demand, and the MVC in the effort discounting task (Supplementary Fig. 7).

R1.6db) "*And why was timepoint T1 not analysed/reported here, as described in the main manuscript text?*"

This is an important point. The section you mention includes two distinct analyses:

1. Linear Mixed Effects Models: We performed these analyses to investigate drug effects on mood (including alertness) and physiological parameters (including systolic blood pressure

and heart rate) across time points (T_0, T_1, T_2). Here, T_0 served as the reference to assess changes induced by the drugs at two distinct time points after drug administration, T_1 (before starting the experimental tasks) and T_2 (after finishing the experimental tasks). The regression models revealed credible biperiden effects on systolic blood pressure and heart rate at both T_1 and T_2, with the strongest effects observed at T_2 (Supplementary Figure 5). Alertness was credibly altered only at T_2 (by both biperiden and haloperidol), but not at T_1 (Supplementary Figure 6).

2. Correlation Analysis: After identifying the variables that were altered by the drug manipulations, we tested if these alterations were linked to drug-induced changes in behaviour, using correlation analyses.

Since alertness was only modulated at T_2, and the modulation of systolic blood pressure and heart rate reached its strongest effects at T_2 (s. Supplementary Figure 5), we focused the subsequent control analysis on the change between T_0 (baseline) and T_2. However, we recognize that readers may also be interested in correlations between computational parameters and changes in physiological measure at T_1, especially given the potential influence of differing drug peaks on this relationship. Therefore, we now include additional analyses on the changes between T_1 and baseline (for systolic blood pressure and heart rate) (supplementary results, lines 82 - 91). To stick with the Bayesian framework, we used Bayesian correlation instead of Pearson correlation analyses, which, in line with the results from the original analysis, revealed no substantial evidence for credible correlations between changes in physiological and mood measures and drug-induced behavioural changes at any timepoint. We have also added a clarification to the methods section (lines 910 - 912) and now present findings regarding timepoint T_1 in the supplementary material (supplementary results, lines 109 - 124 and supplementary methods, lines 295 - 300). These sections now read as follows:

Methods:

> Specifically, we calculated the absolute differences in mood ratings and physiological parameters between T_0 (before drug administration) and the time point where a significant drug effect was observed (either T_1 or T_2).

Supplementary methods:

> Importantly, to test whether credible changes in physiological parameters or mood ratings induced by drug administration could explain changes in behaviour, we used

Bayesian correlation tests to examine possible associations. We correlated difference values (T_0 vs. timepoints with credible drug-induced influences; i.e., T_2 for mood ratings, T_1 and T_2 for physiological parameters) with the mean estimates of all shift parameters that were credibly modulated by either haloperidol or biperiden.

Supplementary results:

We found no credible correlation between any computational parameter that was modulated by biperiden and changes in systolic blood pressure at T_1 in the effort (r = 0.10, $HDI_{95\%}$ = [-0.14; 0.33] for $s\kappa_{BIP}$; r = -0.16, $HDI_{95\%}$ = [-0.39; 0.07] for $s\beta_{BIP}$) and delay (r = 0.06, $HDI_{95\%}$ = [-0.19; 0.29] for $s\beta_{BIP}$) discounting task. Similarly, we did not find any credible correlation between the computational parameters and biperiden-induced changes in systolic blood pressure at T_2 (r = -0.04, $HDI_{95\%}$ = [-0.27; 0.20] for $s\kappa_{BIP}$ (effort); r = -0.08, $HDI_{95\%}$ = [-0.31; 0.17] for $s\beta_{BIP}$ (effort); r = 0.13, $HDI_{95\%}$ = [-0.10; 0.35] for $s\kappa_{BIP}$ (delay)). The same applies for biperiden-induced reductions in heart rate at T_1 (r = 0.05, $HDI_{95\%}$ = [-0.18; 0.28] for $s\kappa_{BIP}$ (effort); r = 0.06, $HDI_{95\%}$ = [-0.18; 0.28] for $s\beta_{BIP}$ (effort); r = -0.06, $HDI_{95\%}$ = [-0.29; 0.18] for $s\kappa_{BIP}$ (delay)) and at T_2 (r = 0.23, $HDI_{95\%}$ = [-0.00; 0.45] for $s\kappa_{BIP}$ (effort); r = -0.09, $HDI_{95\%}$ = [-0.32; 0.16] for $s\beta_{BIP}$ (effort); r = -0.18, $HDI_{95\%}$ = [-0.40; 0.07] for $s\kappa_{BIP}$ (delay)) for both experimental paradigms. Alertness, which was affected by both drugs at T_2, also did not show any credible correlations with biperiden- (r = 0.14, $HDI_{95\%}$ = [-0.10; 0.37] for $s\kappa_{BIP}$ (effort); r = 0.77, $HDI_{95\%}$ = [-0.16; 0.30] for $s\beta_{BIP}$ (effort), r = -0.16, $HDI_{95\%}$ = [-0.38; 0.08] for $s\beta_{BIP}$ (delay)) as well as haloperidol-induced changes (r = -0.05, $HDI_{95\%}$ = [-0.28; 0.19] for $s\kappa_{HAL}$ (effort); r = -0.09, $HDI_{95\%}$ = [-0.33; 0.16] for $s\beta_{HAL}$ (effort); r = 0.05, $HDI_{95\%}$ = [-0.20; 0.29] for $s\kappa_{HAL}$ (delay)).

Reviewer #2:

"*The authors tested the effects of two drugs, one dopaminergic (haloperidol) and one cholinergic (biperiden) on choices made by healthy volunteers in two tasks, one testing effort discounting and the other delay discounting of monetary rewards. The main results are opposite effects on effort discounting, the willingness to exert effort being decreased by haloperidol and increased by biperiden, plus a single effect of haloperidol on delay discounting.*

To my knowledge, this precise combination of 2 tasks by 2 drugs has never been reported. The opposite effects on effort discounting are novel and interesting. The manuscript is clearly written, the analyses are sophisticated. The authors should be commended for the impressive set of physiological and self-report data that were collected to document drug effects."

We thank you for your positive overall evaluation of our manuscript and your thoughtful comments and suggestions for clarification. We are grateful for the acknowledgment of our study's novelty and the thoroughness of our analysis and data collection.

R2.1) "*The comparison between the two tasks is problematic. I noted that they are analyzed separately, but there is still an implicit comparison in the conclusions, for instance when stating that biperiden affects effort but not delay discounting, or that dopamine is domain-general and acetylcholine domain-specific. There are several reasons why the tasks cannot be compared: one was always done after the other (hence at different latencies relative to the peak of drug efficiency), they employed different amounts of reward, one option was fixed in one task and not in the other, choices were hypothetical in one task and not in the other, etc. As is clearly apparent in Fig. 2, choice behavior was quite different: while choices were equally sensitive to effort and reward in the first task, they were almost insensitive to delay and dominated by reward in the second task. These design issues cannot be changed, but the comparison can be avoided, for instance by focusing on effort discounting, which yielded the more convincing results."*

We fully agree with your comments regarding task comparison. You are correct that the methodological differences between the tasks preclude any direct formal comparisons between effort and delay discounting and that findings in the effort discounting task are more convincing. We have carefully revised the discussion section in the manuscript to remove any sections that imply formal comparisons, while maintaining our original findings (see lines 373 - 377 and 601 - 603).

In short, we found that reducing dopaminergic transmission at D2-like receptors decreased participants' willingness to invest physical effort for monetary rewards and attenuated the impact of delay on decision making. In contrast, cholinergic M1 receptor manipulations promoted the preference for high-effort options, without affecting delay-based choices.

and

Our findings indicate that, while D2 receptor activity plays a role in integrating both delay and effort costs, acetylcholine, acting at M1 receptors, may have a more specific role in effort processing.

Moreover, we now acknowledge limitations arising from the different experimental designs and explicitly discuss these issues in the discussion section of the manuscript (see lines 564 - 577):

Lastly, it is important to note that several factors preclude a direct quantitative comparison between the delay and effort discounting tasks. These include the distinct nature of rewards and costs (hypothetical vs. real), varying reward magnitudes in both tasks (large vs. small), and differences in task structure (fixed vs. variable alternative option). Additionally, both tasks were consistently performed in a fixed order within and between participants (effort followed by delay). While this ensured consistent drug levels within each task, it is potentially introducing task order effects and possibly leading to different drug concentrations between tasks. These methodological differences limit our ability to directly compare drug effects across the two cost domains. Future research would benefit from applying experimental paradigms that manipulate both delay and effort costs within the same task, allowing a more controlled and direct comparison of how pharmacological manipulations differentially influence sensitivity to these distinct cost types.

The manuscript further includes a correlational analysis between model-estimated effort and delay discounting parameters (results, lines 350 - 363), because we believe this provides valuable insights into potential relationships between discounting across the two cost domains. Of course, you are right that we cannot directly compare the absolute magnitude of discounting between the tasks – i.e., it precludes us from making statements like "people show stronger delay discounting than effort discounting". Nevertheless, as both parameters are assumed to validly capture the construct of "discounting" (sensitivity towards effort or delay), we would argue that correlation analyses are still perfectly warranted: if there is a relationship between delay and effort discounting, then participant A who shows stronger delay discounting

compared to participant B – independent of the absolute magnitude of either the delay or effort discounting. However, if you disagree with our reasoning, we are also open to removing it from the manuscript, as this analysis is by no means central to our main findings.
Please note that, to maintain a consistent analysis approach (as also mentioned by reviewer 1 in comment #R1.1d), we now use a Bayesian correlation analysis, which confirms the original Pearson correlation findings, showing no significant correlation between discounting behaviour in the two domains. The new sections in the methods (lines 926 - 929) and results section (lines 356 - 360) now read as follows:

Methods:

> To examine this, we performed a Bayesian correlation analysis using the mean estimates of the κ parameters from the placebo conditions in both the effort and delay discounting task.

Results:

> In line with previous findings (Klein-Flügge et al., 2015; Seaman et al., 2018), we found no relationship between individuals' tendency to discount rewards based on efforts compared to delays (no credible correlation between the effort and delay discounting parameters κ under placebo; r = -0.17, $HDI_{95\%}$ = [-0.06; 0.39]).

Importantly, by measuring both effort and delay discounting within the same participants and pharmacological conditions, we aimed to provide a broader perspective on how these distinct aspects of cost-benefit decision making may be differentially influenced by dopaminergic and cholinergic manipulations. While our experimental design does not allow direct comparisons between the tasks, we still investigated the potential neuropharmacological influences within each cost domain. Please note that our statement regarding biperiden affecting effort but not delay discounting was intended purely as a descriptive summary of our observed (modelling- and regression-based) results *within* each task, without implying a direct statistical comparison between the two.
We hope that this approach is now clarified in the revised manuscript.

R2.2) "*The parameters that capture drug effects in choice models seem arbitrary. As only discount factors and choice stochasticity can change with drug conditions, the model fit would miss an effect on reward sensitivity, which may be expected given the model-free results and the literature on dopamine. It would also miss a change in the global willingness to accepts costs, irrespective of reward, effort or delay levels. My suggestion is to include a weight on*

reward in the value function and an intercept (bias) in the choice function, with a potential modulation of these two parameters by drug conditions. To preserve recoverability, some parameters could be common to the two tasks (e.g., inverse temperature)."

We appreciate your thoughtful suggestion and agree that considering reward sensitivity and a choice bias would be valuable extensions to the model. During our study design phase, we thoroughly discussed this possibility and consulted with colleagues working in this area. While we used regression models to test the direct effects of task manipulations on choice behaviour, i.e., the impact of reward (reward sensitivity) and costs (cost sensitivity) on decisions and the general willingness to choose high-cost options, our primary goal of the computational model was rather to capture individual discounting tendencies with the parameter κ. This parameter integrates the interplay between costs and rewards within the decision-making process and thus serves as a summary statistic that captures general discounting behaviour. We had explored the inclusion of additional parameters capturing reward and choice bias. However, these parameters capture overlapping variance in the data, which we believe is the reason this led to severe convergence issues and unreliable parameter estimates. This is also in agreement with the broader literature on hierarchical Bayesian modelling of discounting behaviour, where reward sensitivity and bias parameters are commonly not used within similar frameworks. Thus, we eventually followed the approach set by established studies using similar hierarchical Bayesian models that analysed discounting behaviour under different conditions (Wagner et al., 2020, J. Neurosci; Knauth & Peters 2022, Psychophysiology; Mathar et al., 2023, PLOS Comput. Biol).

R2.3a) "*One the main objectives announced in the introduction is to disambiguate the role of dopamine in delay-based decision-making. This cannot be done by using the same type of task as was used in previous studies. What the authors mean is that they intend to add another dataset to an already large literature on dopamine and delay discounting* "

It is absolutely correct that our study design does not allow us to "disambiguate the role of dopamine in delay-based decision making" – we realize this wording is neither appropriate nor what we meant to convey. We have therefore revised the relevant sections in the introduction (lines 102 - 104) and discussion (lines 383 - 385) section of the manuscript to avoid overgeneralizations and now emphasize that our study makes a contribution by providing a new set of data using a large sample size and a within-subjects design, which we hope will allow for more robust conclusions.

Introduction:

Third, we sought to contribute new evidence to the thus far conflicting literature on the role of dopamine in delay-based decision making using a large sample size and a within-subjects design.

Discussion:

Third, we aimed to address the conflicting literature on dopamine's role in delay-based decision making by providing data from a large sample using a within-subjects design.

R2.3b) "*Besides, the authors should refrain from concluding about dopamine (or acetylcholine) in general, since they used drugs that are specific to a subtype of dopamine (or acetylcholine) receptors.*"

You are absolutely correct that our pharmacological manipulations targeted specific receptor subtypes. We fully agree that it is important to avoid overgeneralizations and be more nuanced about the roles of dopamine or acetylcholine. We have carefully revised several sections of the manuscript to make sure that our interpretations reflect our findings. For example, we have revised following sections in the abstract (lines 33 - 35), results (lines 263 - 265), and the discussion (lines 373 - 377):

Abstract:

Together, our data suggest that dopamine, acting at D2 receptors, modulates both effort and delay discounting, while acetylcholine, acting at M1 receptors, appears to exert influence on effort discounting only.

Results:

This provides strong evidence for a credible modulation of effort discounting by cholinergic M1 and dopaminergic D2 receptor manipulation, despite the slight overlap.

Discussion:

In short, we found that reducing dopaminergic transmission at D2-like receptors decreased participants' willingness to invest physical effort for monetary rewards and attenuated the impact of delay on decision making. In contrast, cholinergic M1 receptor manipulations promoted the preference for high-effort options, without affecting delay-based choices

R2.4) "*The 6 possible effects (main effect, reward sensitivity and cost sensitivity for the 2 tasks) should be illustrated in Fig. 3, to avoid the impression that results are arbitrarily picked.*"

We appreciate your suggestion to enhance the clarity of Figure 3. To ensure full transparency we have now updated Figure 3 to include the two previously omitted plots (see page 61).

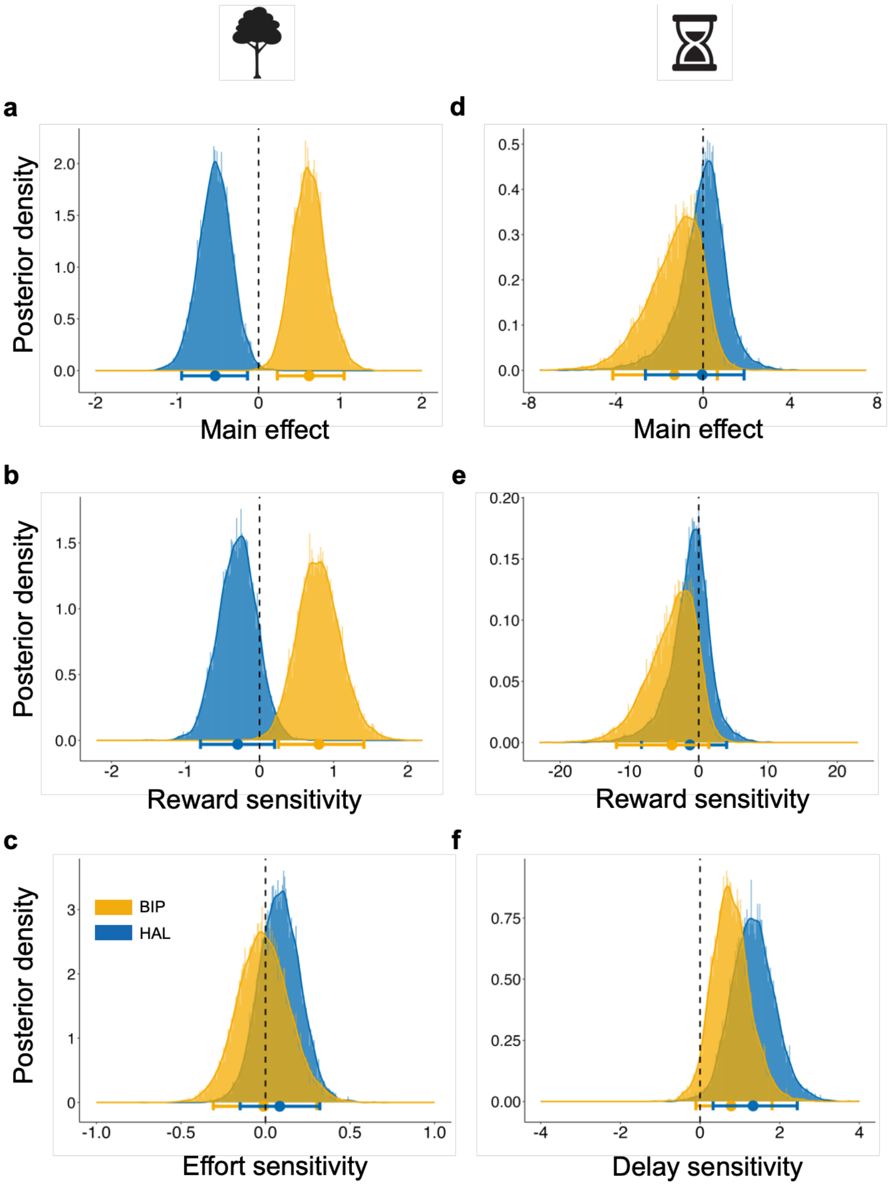

Fig.R2 (Fig.3) Drug Effects on Choice Behaviour. Posterior distributions and 95% HDI of the Logistic Bayesian Generalized Linear Mixed Models depict the estimate of each effect on choosing the high-cost option. (**a**) Biperiden credibly increased the overall willingness to invest physical effort for a corresponding reward, while haloperidol had the opposite effect. (**b**) Biperiden increased reward sensitivity in the effort discounting task, as indicated by a biperiden-by-reward interaction, without a credible effect of haloperidol. (**c**) In contrast, neither drug affected effort sensitivity (**d**) In the delay discounting task, the willingness to tolerate delays for rewards was not affected by either drug. (**e**) Likewise, neither drug modulated reward sensitivity in the delay discounting task. (**f**) However, haloperidol decreased the delay sensitivity, with no credible effect of biperiden. Here, a positive estimate of the interaction effect between haloperidol and delay indicates a reduction of the negative parameter estimate associated with delay, suggesting an attenuation of the impact of delay on choice behaviour. Bold dots represent the mean group-level estimate of the posterior distribution. The horizontal bars represent the group-level 95% highest density interval.

R2.5) "*The possible motor effects of dopamine are not taken into account in the effort model, and neither is the potential impact on fatigue related to repeated squeezing. These potential effects can be tested on the parameters of force pulses and their variation across task trials*"

This is an important point regarding potential motor- and fatigue-related effects of the pharmacological conditions in the effort task (also raised by reviewer 1 in comment #R1.4). Unfortunately, we did not record force pulses, which prevents us from following your suggestion to directly investigate motor-related effects.

However, we have now addressed the potential role of fatigue. To this end, we extended the regression models to include *trial number* as a regressor (to examine overall fatigue effects), along with both *drug* x *trial number* interaction terms (to test for any drug-dependent fatigue effects). This analysis revealed a general fatigue effect throughout the task, evidenced by a reduced tendency of choosing the high-effort option as a function of trial number (HDI_{Mean} = -0.60, $HDI_{95\%}$ = [-0.75; -0.46]), as well as a significant interaction between trial number and haloperidol (HDI_{Mean} = -0.43, $HDI_{95\%}$ = [-0.63; -0.23]). This indicates that, in addition to the main effect of haloperidol on choice behaviour, the fatigue effect during the experimental session was even more pronounced under haloperidol compared to placebo. Importantly, adding the new predictors did not change our main findings (main effect of both drugs in the effort discounting task; credible delay x haloperidol interaction in the delay discounting task; credible main effects of all reward and cost manipulations). We added this new analysis to the main manuscript (results, lines 186 - 202).

> Having established distinct task-specific drug effects on participants' choice behaviour, we next investigated potential confounding order and fatigue effects using separate GLMMs for effort- and delay-based decision making. These models mirrored the previously described GLMMs but included additional predictors to test for fatigue and session effects and their modulation by drugs.
>
> To test for possible fatigue effects, we added trial number as well as the two-way interaction with drug as additional predictors. This analysis revealed a credible main effect of trial number for both the effort and delay discounting task (trial number effect on effort discounting: HDI_{Mean} = -0.60, $HDI_{95\%}$ = [-0.75; -0.46]; trial number effect on delay discounting: HDI_{Mean} = -0.20, $HDI_{95\%}$ = [-0.33; -0.08]), suggesting that the tendency to choose high-cost options, irrespective of cost type, decreased over the course of the experiment. Notably, we found a credible interaction effect between trial number and haloperidol in the effort discounting task (HDI_{Mean} = -0.43, $HDI_{95\%}$ = [-0.63; -0.23]), indicating that the fatigue effect was more pronounced under haloperidol compared to placebo. Importantly, even after accounting for these fatigue-related

effects, the main effect of haloperidol on effort discounting remained credible (HDI_{Mean} = -0.48, $HDI_{95\%}$ = [-0.90; -0.05]) (Supplementary Table 12 and 13).

While our GLMM approach provides insights into general fatigue effects, we acknowledge that recent studies using computational models have revealed distinct forms of fatigue, which might be modulated differentially by dopaminergic and cholinergic manipulations (Müller et al., 2021, *Nat. Commun*; Matthews et al., 2023, *Cognition*). However, investigating drug effects on these distinct forms of fatigue was beyond the scope of our manuscript. Moreover, these studies used different task designs and did not involve any pharmacological intervention. Additionally, more detailed analyses of motor effects, especially in the context of dopamine, would be valuable. We now discuss these issues in a new paragraph in the discussion section (lines 551 - 564).

Third, as participants were required to exert physical effort on each trial, fatigue effects could have developed in the effort discounting task. While our additional analysis confirmed a general fatigue effect and a dual role of haloperidol in both reducing the overall propensity to invest effort and in exacerbating the fatigue effect throughout the task, we acknowledge that recent studies revealed the existence of distinct states of fatigue (Matthews et al., 2023; Müller et al., 2021). Importantly, this was discovered by using task paradigms and computational models that were designed to distinguish between these different types of fatigue. Additionally, it is important to consider that potential motor effects, particularly in the context of dopaminergic manipulations, could also affect effort discounting behaviour. Future studies could extend our approach by incorporating measurements of force pulses to investigate potential motor-related effects and apply task designs and computational models specifically tailored to capture how drug manipulations might affect different forms of fatigue.

R2.6) "*Session effects and drug-by-session interactions should be included in linear mixed-effect models. There is usually meta-learning with these tasks, such that sensitivity to drug effects might differ between the first and second sessions in within-subject designs.*"

Thank you for your comment regarding potential session effects and drug by session interactions. Especially in the effort discounting task, experience with the handgrip device could change choice behaviour. While our initial analysis focused primarily on isolating drug effects, we acknowledge the importance of accounting for potential learning or drug order effects across sessions.

Therefore, we re-ran our regression models, including *session* as a regressor along with both two-way *drug x session* (i.e., *haloperidol x session* and *biperiden x session*) interactions. This analysis confirmed a main effect of session (HDI_{Mean} = 0.49, $HDI_{95\%}$ = [0.12; 0.85]), indicating increasing preference for high-effort options across sessions, likely reflecting task familiarity. Importantly, the interaction effects between either drug and session were not credible (haloperidol x session: HDI_{Mean} = -0.43, $HDI_{95\%}$ = [-1.02; 0.13]; biperiden x session: HDI_{Mean} = -0.16, $HDI_{95\%}$ = [-0.76; 0.45]), suggesting that session effects on effortful choices were not modulated by either of the two drugs. Importantly, including session and session x drug as predictors did not change our main findings. That is, we still observed the main effects of both drugs in the effort discounting task, a credible delay x haloperidol interaction in the delay discounting task, and credible main effects of all reward and cost manipulations. We have incorporated these additional analyses into the manuscript (results, lines 186 - 211) and the supplementary materials (Supplementary Table 14 and 15).

> Having established distinct task-specific drug effects on participants' choice behaviour, we next investigated potential confounding order and fatigue effects using separate GLMMs for effort- and delay-based decision making. These models mirrored the previously described GLMMs but included additional predictors to test for fatigue and session effects and their modulation by drugs. […] Next, to investigate session and drug-order effects, we included session and the two-way interactions between session and drug as additional predictors. These analyses confirmed a main effect of session only for effort-based choices (HDI_{Mean} = 0.49, $HDI_{95\%}$ = [0.12; 0.85]), suggesting increased preference for high-effort options in later sessions, likely reflecting task familiarity. Crucially, the interaction effects between either drug and session were not credible in both tasks (Supplementary Table 14 and 15). This indicates that neither dopaminergic nor cholinergic manipulations modulated learning effects across sessions. Moreover, this finding underscores that the observed drug-induced effects cannot be explained by drug order effects.

R2.7) “*Results on response times are not bringing any additional insight. They could easily be moved to supplementary material.*”

We agree and have therefore moved the decision time analyses to the supplementary materials to maintain a clear focus on the core findings of our study.

R2.8a) "*The interpretation of haloperidol effects in terms of proximity is purely tautological. Explaining that participants prefer delayed rewards because they are less sensitive to temporal proximity is just paraphrasing the result.*"

We understand that the emphasis on proximity in the context of delay discounting could come across as circular. It is important to clarify that we intended to relate our findings to a theoretical framework described by Soutschek et al. (2023, *Biol Psychiatry Glob Open Sci*). This theory suggests that dopamine plays a dual role in (i) biasing choices towards options with a general proximity advantage and (ii) modulating the computation of acceptable costs in return for rewards.
Our findings in the effort discounting paradigm align with the latter cost-reward computation mechanism, as D2 receptor antagonism shifted behaviour towards low-reward/low-effort options. However, this mechanism does not fully explain the delay discounting task results, where dopaminergic D2 receptor antagonism decreased the sensitivity towards delays, without biasing choices toward low-reward/low-delay options. Because of this, we reasoned that in the delay discounting task, an additional factor – psychological proximity – might play a role. However, we acknowledge that this idea is highly speculative and requires further investigation. As we did not explicitly control for proximity in our task design, we have revised the relevant section to be less definitive and more speculative in tone (see lines 433 - 451).

> In other words, this theoretical framework proposes that dopamine may play a dual role in (i) promoting choices towards options with a psychological proximity advantage and (ii) weighing reward magnitudes against associated costs. Our findings of decreased delay discounting under haloperidol align with this theory, potentially suggesting a diminished preference for more proximal options when D2-receptor activity is reduced, resulting in increased endurance for higher (time) costs. On the other hand, the reduction of dopaminergic neurotransmission had the opposite effect on effort discounting, leading to a diminished willingness to bear higher (effort) costs. This apparently conflicting finding between effort and time costs may be explained by the fact that neither the low-effort nor the high-effort option had a pronounced psychological proximity advantage. Thus, in this specific context of cost-benefit decision making, the proximity factor appears to be less relevant, and dopamine instead may promote behaviour aimed at maximizing rewards simply by weighing rewards against associated costs. Importantly, our experimental paradigm did not directly manipulate psychological proximity, nor did it distinguish between proximity effects and the impact of delay. Therefore, specific effects of dopamine on psychological proximity in human delay discounting remain highly speculative and

warrant further investigation with experimental paradigms specifically tailored to this question.

R2.8b) "*Besides, it is incoherent to state that dopamine is domain-general because it has a role in processing effort in one case and proximity in the other. If the role of dopamine is different in effort and delay discounting, then it is not domain general.*"

While the term "domain-general" referred to our finding that dopaminergic manipulations modulated both cost factors (irrespective of the direction), we agree that the term might be too broad of a conclusion, especially given that we tested only two cost factors. For this reason, we have now revised corresponding sections in the abstract (lines 33 - 35), the introduction (lines: 97 - 102), and the discussion (lines 601 - 603) to avoid this term and instead focus on our specific findings.

Abstract:

Together, our data suggest that dopamine, acting at D2 receptors, modulates both effort and delay discounting, while acetylcholine, acting at M1 receptors, appears to exert a more specific influence on effort discounting.

Introduction:

Second, we aimed to assess the contribution of acetylcholine to cost-benefit decision making and conceptually contrast it with the effects of dopamine. In doing so, we investigated whether any effects of muscarinic or dopaminergic receptor antagonism would modulate the computation of both time and effort costs or have a more specific effect on one cost dimension.

Discussion:

Our findings indicate that, while D2 receptor activity plays a role in integrating both delay and effort costs, acetylcholine, acting at M1 receptors, may have a more specific role in effort processing.

Reviewer #3:

"*In this manuscript the authors studied the effect of dopaminergic and muscarinic antagonists on effort discounting and time discounting using two separate cognitive tasks. They used a within person design in which each participant performed both tasks in three separate occasions, receiving one of the three possible treatments (placebo, haloperidol, or biperiden) on each occasion. The authors analyzed the behavioral data using a GLMM approach and a computational modelling approach. They report that haloperidol and biperiden had opposite effects on the effort discounting task, with haloperidol decreasing and biperiden increasing the willingness to make effort to gain rewards. For the delay discounting, they found that only haloperidol had an effect by decreasing delay discounting whereas biperiden had no effect.*

There are not many studies in humans contrasting the effects of manipulations of the dopaminergic and acetylcholinergic systems within the same participants. In this sense, these are interesting data that increases our understanding on how dopamine and acetylcholine modulate decision-making processes. However, I believe that the data is less clarifying on the role of these neurotransmitters as the authors imply in the manuscript and the presentation would benefit from more clarity throughout, especially when it comes to the results section that necessarily needs to include a brief description of the methods."

We thank you for your thoughtful comments on how to improve clarity in our manuscript, particularly within the results section. We have carefully revised these sections to incorporate the necessary details and ensure that our findings are presented in a clear manner. We detail the specific changes that were applied below in response to each comment.

R3.1) "*It is not clear why figures 3 and 6 do not include the posterior distribution of the placebo effect. I understand that figure 4, showing the effects of the drugs on the model parameters, do not include it because the estimated parameters are a shift from the parameter of interest under placebo. However, I understand that the GLM employed to estimate the main effect of drug and the interaction with reward sensitivity or delay discounting is also estimated for the placebo effect. By looking at the behaviroal data in figure 2, it seems that neither drug will differ from the placebo. Even if the authors can still claim that haloperidol and biperiden have opposite effects, I think it is misleading not to depict the placebo effect."*

We believe that there may be a misunderstanding and we acknowledge that we did not write this paragraph with the necessary clarity. In our regression models, the drug condition (placebo, haloperidol, biperiden) was included as a 3-level categorical predictor with placebo as the reference. This means that there is no inherent *effect* of placebo, and effects plotted in

Figures 3 and 6 represent the estimated drug effects (for haloperidol and biperiden) *in reference to the placebo condition*. To make this important point more explicit, we have added the following paragraph to the methods section (lines 747 - 753):

> In these models, we regressed choices (choosing the high-cost option vs. choosing the low-cost option) on fixed-effect predictors including drug, reward, cost (i.e., delay or effort), and all possible interaction terms. Importantly, to test drug-specific effects on behaviour, we included the drug condition (placebo, haloperidol, biperiden) as a 3-level categorical predictor with placebo set as the reference. Thus, all drug-related main and interaction effects are estimated in reference to the placebo.

If we understand correctly, you are interested in visualizing the choice behaviour specifically under the placebo condition. To address this, we have re-run the regression analyses using only the data from the placebo session. This captures the main effects of task parameters (e.g., reward and cost) under the (baseline) placebo condition. These results are now included in the supplementary materials (supplementary results; see lines 56 - 63; and Supplementary Figure 1), reproduced below for convenience.

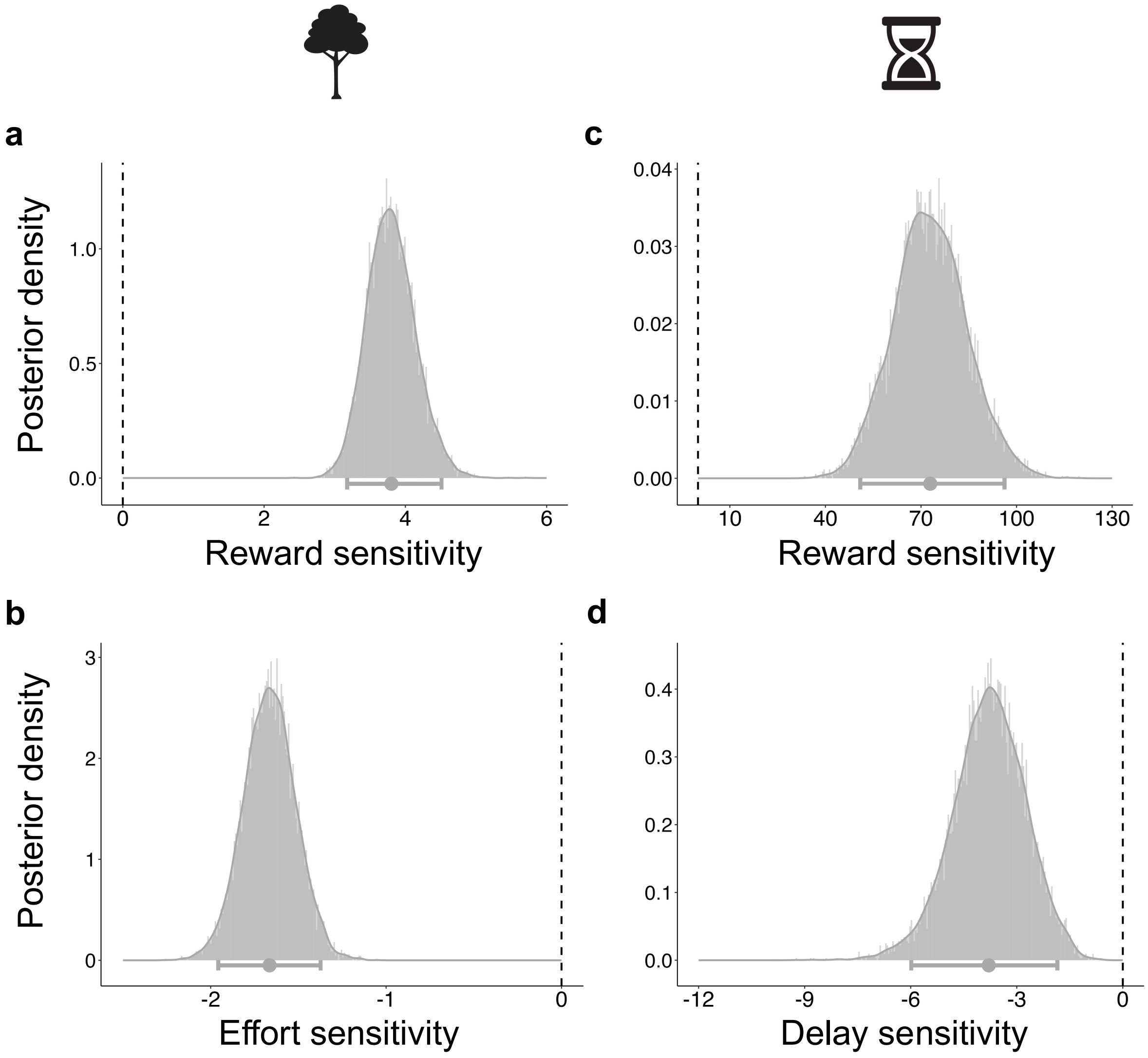

Supplementary Fig. R3 (Fig S1). Choice Behaviour in the placebo (baseline) condition. Posterior distributions and 95% HDI of the Logistic Bayesian Generalized Linear Mixed Models depict the estimate of each task parameter on choosing the high-cost option. (**a**) Higher reward magnitudes increased the overall willingness to invest physical effort for a corresponding reward in the effort discounting task. (**b**) Higher levels of effort had the opposite effect. (**c**) Similarly, higher reward magnitudes increased the likelihood to choose the high-cost option in the delay discounting task. (**d**) In contrast, higher levels of delay decreased the willingness to choose the high-reward/high-delay option. Bold dots represent the mean group-level estimate of the posterior distribution. The horizontal bars represent the group-level 95% highest density interval.

The supplementary results now read as follows:

> To analyse choice behaviour in the absence of drug manipulations, we conducted Logistic Bayesian Generalized Linear Mixed Models exclusively focusing on the data from the placebo (baseline) session. This analysis confirmed credible main effects of each task parameter on choice behaviour, demonstrating the expected patterns of reward and cost sensitivity across both tasks. Specifically, in both tasks, increased reward magnitudes were associated with a higher likelihood of choosing the high-cost option, while increased effort or delay levels decreased this likelihood (Supplementary Fig. 1 and Supplementary Table 4 and 5).

It is important to note that Figure 2, while providing a descriptive overview of choice behaviour as a function of either reward or cost levels, does not reveal the orthogonal contributions of the different predictors, and their interactions, which the regression model accounts for. While visually the differences compared to placebo might seem marginal, the regression model revealed statistically credible drug effects when controlling for the interplay of costs and rewards.

R3.2a) "*Related to the comment above, the descriptions of the GLMs need important details such as how the different regressors were specified. It is not clear whether the authors controlled for order effects of the treatment manipulation.*"

Thank you again for pointing us to the need for greater clarity in our description of the GLMMs. The choice of predictors in our original models was hypothesis-driven, focusing on the impact of drug manipulations on reward and cost sensitivity. However, as other reviewers also highlighted, it is also crucial to account for other potential confounding effects.
We have conducted additional regression-based analyses incorporating *trial number* (as a measure of fatigue) and *session* (as a measure for session and drug-order effects) as additional predictors in separate regression models, along with their interactions with the different drug conditions. These analyses provide insights into possible fatigue effects across trials as well as any drug order and session effects. This revealed a main effect of trial number in both the effort and delay discounting task (effort discounting task: HDI_{Mean} = -0.60, $HDI_{95\%}$ = [-0.75; -0.46]; delay discounting task: HDI_{Mean} = -0.20, $HDI_{95\%}$ = [-0.33; -0.08]), suggesting a general fatigue effect throughout both tasks. Importantly, we also found a credible interaction between trial number and haloperidol in the effort discounting task (HDI_{Mean} = -0.43, $HDI_{95\%}$ = [-0.63; -0.23]). This indicates that under haloperidol, the fatigue effect during the experimental session was more pronounced compared to placebo, indicating a dual role of haloperidol in reducing the willingness to invest effort and exacerbating the fatigue effect throughout the effort task. Next, including session as a regressor along with both two-way drug x session interactions confirmed a main effect of session in the effort discounting task (HDI_{Mean} = 0.49, $HDI_{95\%}$ = [0.12; 0.85]), indicating increasing preference for high-effort options across sessions, likely reflecting task familiarity. Importantly, the interaction effects between either drug and session were not credible (haloperidol x session: HDI_{Mean} = -0.43, $HDI_{95\%}$ = [-1.02; 0.13]; biperiden x session: HDI_{Mean} = -0.16, $HDI_{95\%}$ = [-0.76; 0.45]), suggesting that session effects on effortful choices were not modulated by either of the two drugs. Further, this analysis did not reveal any credible session effects in the delay discounting task. Importantly, adding the new predictors did not change our main findings. That is, we still observed the main effects of

both drugs in the effort discounting task, a credible delay x haloperidol interaction in the delay discounting task, and credible main effects of all reward and cost manipulations. The results of these analyses are now presented in the main manuscript (results, lines 186 - 211):

> Having established distinct task-specific drug effects on participants' choice behaviour, we next investigated potential confounding order and fatigue effects using separate GLMMs for effort- and delay-based decision making. These models mirrored the previously described GLMMs but included additional predictors to test for fatigue and session effects and their modulation by drugs.
>
> To test for possible fatigue effects, we added trial number as well as the two-way interaction with drug as additional predictors. This analysis revealed a credible main effect of trial number for both the effort and delay discounting task (trial number effect on effort discounting: HDI_{Mean} = -0.60, $HDI_{95\%}$ = [-0.75; -0.46]; trial number effect on delay discounting: HDI_{Mean} = -0.20, $HDI_{95\%}$ = [-0.33; -0.08]), suggesting that the tendency to choose high-cost options, irrespective of cost type, decreased over the course of the experiment. Notably, we found a credible interaction effect between trial number and haloperidol in the effort discounting task (HDI_{Mean} = -0.43, $HDI_{95\%}$ = [-0.63; -0.23]), indicating that the fatigue effect was more pronounced under haloperidol compared to placebo. Importantly, even after accounting for these fatigue-related effects, the main effect of haloperidol on effort discounting remained credible (HDI_{Mean} = -0.48, $HDI_{95\%}$ = [-0.90; -0.05]) (Supplementary Table 12 and 13).
>
> Next, to investigate session and drug-order effects, we included session and the two-way interactions between session and drug as additional predictors. These analyses confirmed a main effect of session only for effort-based choices (HDI_{Mean} = 0.49, $HDI_{95\%}$ = [0.13; 0.85]), suggesting increased preference for high-effort options in later sessions, likely reflecting task familiarity. Crucially, the interaction effects between either drug and session were not credible in both tasks (Supplementary Table 14 and 15). This indicates that neither dopaminergic nor cholinergic manipulations modulated learning effects across sessions. Moreover, this finding underscores that the observed drug-induced effects cannot be explained by drug order effects.

R3.2b) *"Further, the details on the fact that the GLM on choice data is a logistic regression are only given when presenting the GLM on response times (page 31)!"*

You are correct that we should explicitly state the use of a logistic regression model for our regression-based results. We have added this information at the beginning of the results

section (lines 139 - 142) and methods section (lines 744 - 747). The new sections read as follows:

Results:

> For each of the two tasks, we used a Logistic Bayesian Generalized Linear Mixed Model (GLMM) to investigate the impact of drug manipulation, changes in task parameters, and the interaction between both, on choosing the high-cost option.

Methods:

> To investigate the influence of drug administration (compared to placebo) and experimental manipulations (i.e., reward magnitude, delay, and/or effort) on choice behaviour, we employed Logistic Bayesian Generalized Linear Mixed Models, using the brms package (Bürkner, 2017) in R (Version 4.1.3).

R3.3) "*The authors should try to summarize the analysis that they have done in the results section. For example, on page 7, they could state which models they have tried, and which are eventually selected, with a brief description of the selected models. Similarly, the regression models are not very clearly outlined in the results section. Moreover, the methods section of the modelling should include some basic information like the parameter ranges and how these constraints are implemented. For example, in equation 1, I assume that k is exponentiated to ensure a constraint so that it is >0 when fitting a parameter with a gaussian prior. But this information is not provided, and this exponent may lead to confusion.*"

We have followed your suggestion to enhance the clarity of our model descriptions in the results section. We have revised this section to provide a short summary of the computational model selection process and the choice of predictors in our regression analyses (results, lines 142 - 149 and lines 224 - 232). For more comprehensive details, we refer the reader to the methods section and supplementary material. The new sections read as follows:

New paragraph in the GLMM section:

> Specifically, to regress participants' choices, we included the following fixed-effect predictors: drug condition, reward magnitude, cost level (i.e., effort or delay), and all possible interactions. In the effort discounting task, we used the difference terms (high-cost option minus low-cost option) for both reward and effort. For the delay discounting task, we used the absolute reward and delay levels of the varying high-cost option. To

account for within-subject variability, we included random intercepts for each subject along with random slopes for all fixed-effect predictors (see Materials and Methods).

New paragraph in the computational modelling section:

To begin with, we determined the best-fitting discounting models for both effort and delay, comparing four commonly used models (linear, parabolic, hyperbolic, exponential). In line with previous findings (Green & Myerson, 2004; Hartmann et al., 2013; Kirby & Maraković, 1995; Klein-Flügge et al., 2015; Lockwood et al., 2017), model comparisons revealed that effort discounting was best described by a parabolic model, suggesting a greater impact of changes in high rather than low effort levels. In contrast, delay discounting was best described by a hyperbolic model, indicating a greater impact of changes in low rather than high delay levels (Supplementary Table 3).

You are correct that we ensure the discount factor κ is constrained to be positive. The specific methods for achieving this constraint differ slightly between the effort and delay discounting tasks. For modelling effort discounting (using a parabolic model), we use a uniform prior with a range of 0 to 5 and further constrain κ within the code by specifying a lower boundary of 0 in the parameter section. To accommodate the skewed distribution of the κ-values in delay discounting (using a hyperbolic model), we estimate the parameter in log-space. This transformation guarantees a positive κ value, allowing for a wider range of values to be explored during model fitting.

All information regarding the selection of the priors is already presented in the supplementary material (supplementary methods; page 11). If you prefer, we can move this section into the main manuscript for greater visibility.

R3.4a) "*It is not clear to me whether the authors performed a model selection to test whether the inclusion of the shift parameters accounting for the drugs effects are justified. Here it would be key to test whether the shift on the discounting parameters, on the inverse temperature or on both are justified by the data. After that, if those models are justified, one should proceed making statistical inference on the parameters.*"

We appreciate you raising this important point regarding model selection and hypothesis testing. Similar to our approach in choosing predictors for the regression models (see comment #R3.2a), the choice of our model parameters in the computational modelling was

hypothesis-driven and based on prior research. Specifically, we asked whether biperiden and haloperidol affected discounting and choice stochasticity, which is reflected in the two free model parameters, κ and β. Therefore, we included separate shift parameters for each parameter in both models.

To address your concern about model justification, we have now conducted additional analyses to evaluate how adding new parameters changes model performance. We re-ran the models for both the effort discounting and delay discounting task under different model parameters, using four models:

Full Model: The original model with both κ-shift and β-shift parameters.
No Shift Model: Model without any shift parameters.
Only K-Shift Model: Model that includes only the κ-shift parameter.
Only Beta-Shift Model: Model that includes only the β-shift parameter.

For each task, we compared the models using the Leave-One-Out Information Criterion (LOOIC) to identify the best-fitting model. The results, presented below, reveal that the full model, as presented in the manuscript, outperformed the reduced models in both tasks.

**Model**	***Effort***	***Delay***
	**LOOIC**	**LOOIC**
Full	13965.4*	24380.3*
No Shift	15637.6	26294.9
Only k-Shift	14264.5	25630.2
Only beta-Shift	14751.6	24817.2

If you prefer, we are happy to include this section in the supplementary material.

R3.4b) *“Moreover, the key effects of the drugs on the model parameters reflecting effort discounting are just marginally significant. The authors acknowledge that, but the whole manuscript and abstract are written as if these effects were fully significant.”*

Regarding the statistical significance of the κ-shift parameters in the effort discounting task, you are correct that the 95% HDI indeed overlaps with zero. However, our key findings are not only based on the computational modelling, but also on the GLMM results, which provide credible evidence for opposing effects of dopaminergic versus cholinergic modulation on effort-based choices, which aligns with both our initial hypothesis and the direction of change for the κ-shift parameter. Moreover, while the 95% HDI for both κ-shift parameters overlap with zero, the posterior densities still suggest a high degree of belief/confidence (more than 94%) that the parameter is different from zero. This highlights the advantage of Bayesian methods, which offer a less strict interpretation of model parameters by providing a continuous measure of credibility, rather than a strict binary classification offered by frequentists statistics. We would therefore argue that credible effects in the GLMM in combination with these modelling results justify the interpretation of the presence of effects in manuscript and abstract. Nonetheless, we completely agree that a more cautious wording is warranted for the modelling results. We have revised the manuscript to avoid overly strong statements regarding the results from the computational model. Instead, we highlight the complementary evidence provided by the GLMMs, which reinforce the observations of the computational models.

For instance, we revised sections of the abstract (lines 26 - 30), results (lines 310 - 314), and discussion (lines 373 - 377). These sections now read as follows:

Abstract:

> We found opposing effects of dopamine D2 (haloperidol) and acetylcholine M1 receptor (biperiden) antagonism on specific components of effort-based decision making in healthy humans: haloperidol decreased, whereas biperiden increased the willingness to exert physical effort.

Results:

> Taken together, the results derived from hierarchical Bayesian modelling are consistent with findings obtained from the GLMMs. Diminishing dopaminergic D2 receptor activity decreases the willingness to invest effort for rewards, possibly by amplifying effort discounting.

Discussion:

> In short, we found that reducing dopaminergic transmission at D2-like receptors decreased participants' willingness to invest physical effort for monetary rewards and attenuated the impact of delay on decision making. In contrast, cholinergic M1 receptor

manipulations promoted the preference for high-effort options, without affecting delay-based choices.

R3.5) "*The authors found an antagonistic relationship between haloperidol and biperiden on the inverse temperature parameter of the effort discounting task, increasing and decreasing stochasticity, respectively. In the discussion, the authors interpret that D2 antagonism may lead to noisier representations of effort costs. However, the same effects could be observed if D2 antagonism leads to noisier representations of the reward values. These possible explanations could be tested using computational modelling.*"

You are correct that the observed increase in choice stochasticity under D2 receptor antagonism could be linked to noisier representations of either effort costs or reward magnitudes. However, since it is not possible to distinguish these possibilities with our data, we removed the speculation about the possible mechanism and instead stick with our findings. We have now revised the relevant sections of the discussion accordingly (see lines 519 - 520).

> Previous studies have linked D2 receptor antagonism with increased choice stochasticity (Eisenegger et al., 2014; Mikus et al., 2022).

R3.6a) "*The authors notice that the literature on dopamine modulation of effort discounting consistently shows that dopamine decreases effort discounting whereas the literature on delay discounting has conflicting results with sometimes dopamine increasing and sometimes decreasing delay discounting. The authors state that one of the goals of their study is to disambiguate this inconsistent picture. However, as they just did one further experiment (seen from the perspective of the existing literature), I cannot see how they can disambiguate the inconsistencies. They can at most provide evidence for one or the other possible direction of effects.*"

It is absolutely correct that our study design does not allow us to "disambiguate the role of dopamine in delay-based decision making" – we realize this wording is neither appropriate nor what we meant to convey. We have therefore revised the relevant sections in the introduction (lines 102 - 104) and discussion (lines 383 - 385) section of the manuscript to avoid overgeneralizations and now emphasize that our study makes a contribution by adding a new dataset to the existing literature, using a larger sample size and employing a within-subjects design.

Introduction:

> Third, we sought to contribute new evidence to the thus far conflicting literature on the role of dopamine in delay-based decision making using a large sample size and a within-subjects design.

Discussion:

> Third, we aimed to address the conflicting literature on dopamine's role in delay-based decision making by providing data from a large sample using a within-subjects design.

R3.6b) *"Moreover, in the discussion, they claim that their result supports the view that dopamine promotes choices towards options with proximity advantage. In the lack of a manipulation of proximity advantage, I do not believe that the data can tell us anything about how proximity advantage is the determinant explaining the conflicting results in the literature. The authors could at most review the previous results and highlight whether this explanation is consistent across previous experiments. But a clear demonstration that this explanation is in place requires directed experimental work."*

Regarding the role of proximity in delay discounting, we recognize that our experimental design cannot directly demonstrate dopaminergic modulation of the proximity bias. Our initial aim was to embed our results within a theoretical framework (proposed by Soutschek et al., 2023, *Biol Psychiatry Glob Open Sci*), suggesting that dopamine may modulate cost-benefit decision making by weighing rewards against associated costs, while also biasing choices towards options with a psychological proximity advantage.

While our findings could be consistent with a reduced proximity bias following D2 receptor antagonism, we fully agree that this interpretation is highly speculative. More definitive conclusions on the role of dopamine on the proximity bias would require a specifically designed experimental paradigm. As also highlighted by reviewer 2 (see comment #R2.8), we agree that we need to emphasize the speculative nature of this interpretation.

We have now revised the relevant sections of the discussion (lines 433 - 451), making the section more speculative in tone and highlighting the need for further research that explicitly and selectively manipulate proximity. The new paragraph reads as follows:

> In other words, this theoretical framework proposes that dopamine may play a dual role in (i) promoting choices towards options with a psychological proximity advantage and (ii) weighing reward magnitudes against associated costs. Our findings of decreased delay discounting under haloperidol align with this theory, potentially

suggesting a diminished preference for more proximal options when D2-receptor activity is reduced, resulting in increased endurance for higher (time) costs. On the other hand, the reduction of dopaminergic neurotransmission had the opposite effect on effort discounting, leading to a diminished willingness to bear higher (effort) costs. This apparently conflicting finding between effort and time costs may be explained by the fact that neither the low-effort nor the high-effort option had a pronounced psychological proximity advantage. Thus, in this specific context of cost-benefit decision making, the proximity factor appears to be less relevant, and dopamine instead may promote behaviour aimed at maximizing rewards simply by weighing rewards against associated costs. Importantly, our experimental paradigm did not directly manipulate psychological proximity, nor did it distinguish between proximity effects and the impact of delay. Therefore, specific effects of dopamine on psychological proximity in human delay discounting remain highly speculative and warrant further investigation with experimental paradigms specifically tailored to this question.
